# Identification of Novel Arachidonic Acid 15-Lipoxygenase Inhibitors Based on the Bayesian Classifier Model and Computer-Aided High-Throughput Virtual Screening

**DOI:** 10.3390/ph15111440

**Published:** 2022-11-20

**Authors:** Yinglin Liao, Peng Cao, Lianxiang Luo

**Affiliations:** 1 The First Clinical College, Guangdong Medical University, Zhanjiang 524023, China; 2 Department of Pharmacy, Union Hospital, Tongji Medical College, Huazhong University of Science and Technology, Wuhan 430022, China; 3 The Marine Biomedical Research Institute, Guangdong Medical University, Zhanjiang 524023, China; 4 The Marine Biomedical Research Institute of Guangdong Zhanjiang, Zhanjiang 524023, China

**Keywords:** ferroptosis, ALOX15, homology modeling, machine learning, virtual screening, molecular dynamic simulation

## Abstract

Ferroptosis is an iron-dependent lipid peroxidative form of cell death that is distinct from apoptosis and necrosis. ALOX15, also known as arachidonic acid 15-lipoxygenase, promotes ferroptosis by converting intracellular unsaturated lipids into oxidized lipid intermediates and is an important ferroptosis target. In this study, a naive Bayesian machine learning classifier with a structure-based, high-throughput screening approach and a molecular docking program were combined to screen for three compounds with excellent target-binding potential. In the absorption, distribution, metabolism, excretion, and toxicity characterization, three candidate molecules were predicted to exhibit drug-like properties. The subsequent molecular dynamics simulations confirmed their stable binding to the targets. The findings indicated that the compounds exhibited excellent potential ALOX15 inhibitor capacity, thereby providing novel candidates for the treatment of inflammatory ischemia-related diseases caused by ferroptosis.

## 1. Introduction

Unlike apoptosis, necrosis, or autophagy, ferroptosis is a newly identified mode of cell death that occurs in RAS-mutated tumor cells [1]. Ferroptosis is typically triggered by the accumulation of intracellular iron, which induces intracellular lipid peroxidation. The accumulation of intracellular reactive oxygen species (one of the critical features of ferroptosis) leads to organelle and biofilm damage and cell death [2,3]. Ferroptosis can efficiently kill many types of tumors. Numerous drug molecules, mainly Erastin and RSL3 [4], and a number of compounds targeting other key pathway targets that induce tumor ferroptosis have been widely used [5]. However, ferroptosis plays an equally critical role in inflammation and injury. The inhibition of ferroptosis can effectively ameliorate ischemic and degenerative tissue damage in vivo [6]. Therefore, targeting the induction or inhibition of ferroptosis in tumor cells is a novel strategy for the targeted therapy of many diseases, particularly cancer and spontaneous tissue damage [7,8,9,10].

The corresponding protease encoded by the ALOX15 gene, arachidonate 15-lipoxygenase, is an oxygenated lipase with both pro-oxidative and esterifying effects that catalyzes the multimerization of polyunsaturated fatty acids (PUFAs) on cell membranes [11]. In 2016, Yang et al. reported that the ALOX enzyme family can promote the onset of cellular ferroptosis. Subsequent cellular-level studies have revealed that ALOX15 promotes ferroptosis in various tumor cells such as HT-1080 and PANC1 cells [12,13]. Thus, the inhibition of ALOX family enzymes, particularly ALOX15, is essential for the treatment of injury and degenerative diseases triggered by ferroptosis. Novel ALOX15 inhibitors with clinical therapeutic implications have been discovered. For example, Ma et al. (2022) discovered that soya sapogenins can improve ischemia-induced myocardial injury [14]. Cepharanthine, a p53-ALOX pathway blocker developed by Gao et al. [15], reduced ischemia-reperfusion injury in the brain. Although several inhibitors of ALOX15 have been identified to date [16,17,18], various inhibitor molecules with strong clinical therapeutic effects remain undiscovered because of the long lead time of the drug development process and the tedious procedures of chemical extraction and synthesis. Therefore, potential inhibitor molecules should be identified from established commercial compounds to inspire a drug screening effort against ALOX15 targets.

With the rapid development of electronic programming technologies, computer-aided drug design (CADD) is widely being used in drug development [19]. Many assignment functions and components that can calculate the binding energy between small molecules and target proteins and predict the possible types of interactions with the target based on the molecular structure have been developed and used with good results in numerous studies [20]. Computer-dependent drug prediction methods are more cost-effective than conventional experimental methods. [21]. Machine learning models have been used frequently for predicting compound libraries because of their high accuracy in addressing the classification and identification problems based on large amounts of data [22]. Classification models developed based on machine learning methods have been applied to solve numerous compound property prediction problems [23,24,25]. Combining machine learning methods in the typical molecular docking process can help identify compounds with drug potential in a targeted manner.

**Figure 1:**
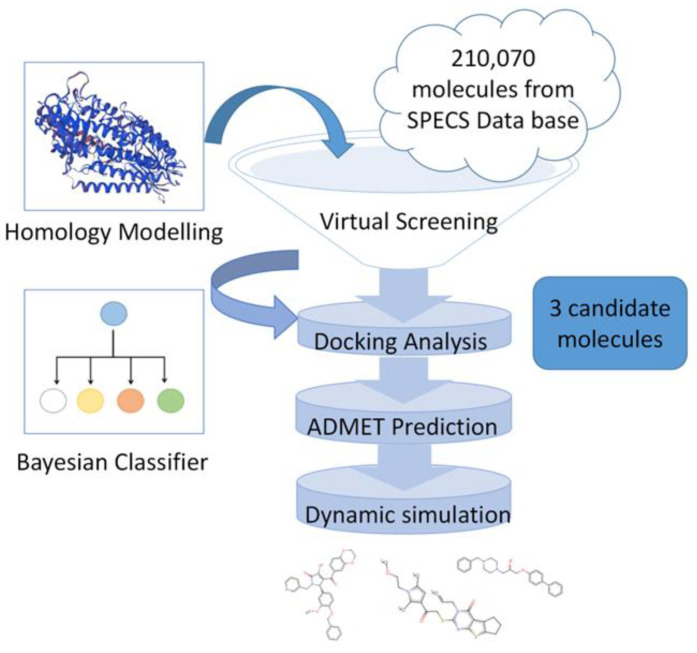
Workflow for this study.

In this study, a coherent computer-aided screening process (Figure 1) was designed to obtain compounds with ALOX15 inhibitory potential from a database of purchasable compounds. The stereospecific protein structure of ALOX15 was established from *Homo sapiens* using a homologous protein modeling approach that combined both structure-based high-throughput screening and Bayesian machine learning models to find three potential compounds. ADMET (absorption, distribution, metabolism, excretion, and toxicity) property prediction and molecular dynamics simulations were used to confirm the drug-forming potential of the candidate compounds. Subsequently, based on existing studies on ALOX15 inhibitors, the molecular features that may play a key role in target binding were analyzed to facilitate the efficient exploration of this protease inhibitor.

## 2. Results

### 2.1. Protein Homology Modeling

In this study, the BLAST search algorithm was used to determine amino acid sequences of proteins with homology to ALOX15 from various biological sources in the UniProt nr_clustered experimental database based on the rabbit-derived ALOX15 amino acid sequence in the PDB database (Table S1). We selected the sequence of ALOX15 derived from *Homo sapiens*, which has 81.12% homology to the PDB parent template sequence. Twenty possible structural models of the ALOX15 protein were constructed based on this sequence, and the best model, M0018, was selected based on an automatically generated evaluation by MODELLER.

**Figure 2:**
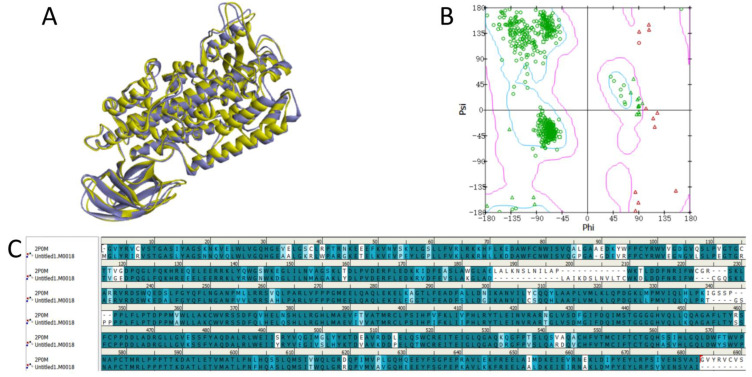
Schematic of the protein homology modeling results: (**A**) structural overlay of homology model M0018 (in yellow) and the template protein (PDB ID: 2P0M, in purple); (**B**) a residue spatial position rationality analysis of the M0018 Ramachandran plot; (**C**) a sequence comparison of the homology model M0018 with the template protein.

The quality of the model was assessed using the profile-3D function. The results revealed that the verified score for M0018 was 278.82, which was considerably higher than the expected low score (136.417). However, this score was close to the expected high score (303.15), which indicated the high quality of the M0018 model (Table 1).The PDB structures of the M0018 and template sequence source proteins were superimposed and the root mean square deviation (RMSD) values of their spatial structures were calculated. To minimize the chance of error resulting from the overlay of various structures, two calculations were performed using the two protein structures as templates. The results are presented in Table 2 and Figure 2A,C. The difference in the spatial position overlap between the two structures was minimal, and the sequence amino acid repeat was sufficiently high. The Ramachandran plots revealed that of the 662 total residues, 87.6% (580) were located in favorable spatial regions, 10.6% (70) in permissive spatial regions, and 1.8% (12) in non-permissive spatial regions, indicating that the protein model was of sufficiently high quality. Therefore, the M0018 protein model was used for the subsequent studies.

### 2.2. Structure-Based, High-Throughput Virtual Screening

The LibDock module of the Discovery Studio platform was used to perform a virtual screening of 210,070 small molecules within the SPECS compound library by using the ALOX15 model based on a homology approach, which resulted in the generation of 46,127 dockable conformations. The top 1% of compounds with the highest LibDock scores (450) were studied based on the assumption that higher scores correspond to superior activity. To avoid a possible chance bias arising from the calculation function of the docking function module, only the molecules that produced two or more interacting conformations with the target were considered in the compound selection.

### 2.3. Chemical Spatial Distribution Analysis

**Figure 3:**
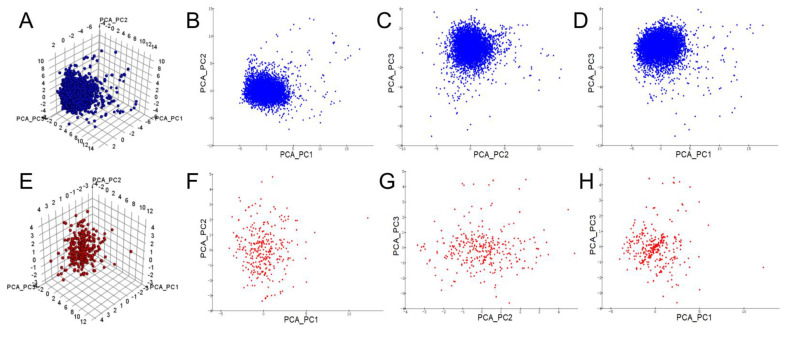
Schematic of the spatial distribution of the principal components (PC1-PC3) of the compounds: (**A**–**D**) chemical spatial distribution of the test set molecules used as model constructs; (**E**–**H**) chemical spatial distribution of the 450 test set molecules from the LibDock screening program.

The discrimination ability of machine learning classification models depends heavily on whether the chemical spatial distribution of the compounds in the training dataset is sufficiently diverse [26]. Therefore, the chemical spatial diversity of 55 calculated descriptors (Table S2) from the entire dataset was investigated using a principal component analysis (PCA) and Tanimoto similarity analysis. The PCA of the molecular descriptors yielded eight key molecular descriptors, namely ES_Count_aasN, ES_Count_aaO, ES_Count_dCH2, Num_AromaticRings, Molecular_FractionalPolarSurfaceArea, IsChiral, Num_Rings, and QED. Five principal components (PCs) were obtained by assigning distinct weighting factors to these descriptors. The first three most critical principal components (PC1–PC3, whose component descriptors and coefficient correlation equations are presented in Table S3) were selected for the chemical spatial analysis. As shown in Figure 3A–D, for the three PCs of the dataset, most of the molecules were clustered in a limited spatial range, probably because of their inherently even descriptor properties and the large number of molecules in the training set. To analyze the chemical similarity between compounds in numerical terms, the fingerprint distance and Tanimoto similarity coefficient (*T*c) between compounds were calculated based on ECFP_2. The results revealed an overall *T*c value of 0.09968 for our training set of molecules, which indicated the overall low chemical structural similarity of the molecules. The same chemical spatial analysis was performed on the test set of 450 LibDock-screened compounds selected from the SPECS compounds. These test set molecules were widely distributed between the three PCs (Figure 3E–H), with a similarity coefficient of 0.13856 calculated from the molecular fingerprints. This result indicated the wide chemical space of these compounds to be screened. Both our training and test sets exhibited a wide distribution of the chemical space, which can be used for machine learning modeling to achieve an important prerequisite for accurate classification.

### 2.4. Machine Learning Classifier Models

**Figure 4:**
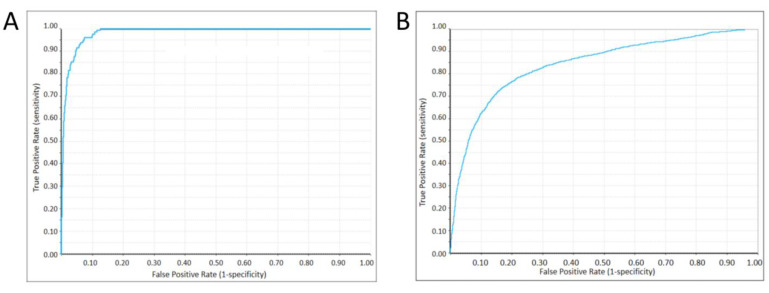
Plots of validated ROC curves for NB models: (**A**) training ROC curve of the model; (**B**) ROC curve obtained from the ten-fold validation of the model.

Bayesian classification models were constructed using 5360 active/inactive ALOX15-targeted compounds obtained from the CHEMBL database. The key molecular descriptors obtained from the dimensionality reduction in the PCA, as well as the molecular fingerprint ECFP_n, were applied to the proposed model construction process. Various fingerprint–descriptor combinations were attempted during the model construction, whereas the area under the ROC curve (AUC) values of the obtained models were considered as indicators representing their classification ability. The best-performing Bayesian model was selected for a further analysis, in which an active flag was used for classification. The naive Bayesian model is based on a binary (i.e., ‘yes or no’) approach to determining whether a compound has target inhibitory activity, with the classification process using the model’s activity cut-off value (−2.366 in our model) as a benchmark, along with a Bayesian score for each compound. When the absolute value of the Bayesian score is greater than the cut-off value, the compound is classified as ‘active’, and the opposite is classified as “inactive” (Table S4). Subsequently, ECFP_6 and eight key descriptors were applied (ES_Count_aasN, ES_Count_aaO, Num_AromaticRings, Molecular_FractionalPolarSurfaceArea, IsChiral, Num_Rings, ES_Count_dCH2, and QED). The training result metrics of this model are presented in Table 3, and its ROC curve is presented in Figure 4A. The Naive Bayesian (NB) model revealed excellent classification recognition ability. For the classification of active molecules, the true positive rate (recall) reached 92.5%; for inactive molecules, the prediction rate of the model for true positives (inactive molecules correctly classified) was 91.3%. The model had an AUC of 0.895, close to 1, which indicated its excellent classification ability. However, combining the tests and the confusion matrix of the active/inactive molecules obtained from the model differentiation (Table 4) revealed that the classifier model was slightly better at identifying inactive molecules than it was at differentiating active molecules. This result implied that more false positive examples may be obtained using the model for classification. However, the risk of incorrectly excluding active compounds can be avoided. Because we used more screening and validation methods throughout the screening process, a slightly higher false positive rate is acceptable.

The constructed Bayesian model was validated using the ten-fold cross-validation method. The results are presented in Table 5, and the ROC plot is displayed in Figure 4B. The ten-fold validation results revealed a similar tendency to the discriminatory ability of the model; that is, the model’s ability to identify inactive molecules is superior to when identifying active molecules. The true positive rates for the classification of active molecules and the discrimination of inactive molecules were 85.5 and 87.7%, respectively. Under the ten-fold validation condition, the classification precision metric of the model was significantly higher for inactive examples than that for active examples (Table 6). Compared with the discriminatory ability recognition metrics of the model, all metrics of the ten-fold model validation results decreased but remained within a good range, which confirmed the reliability of the algorithm used to construct the NB model.

The results obtained by the model in the 10-fold validation test were significantly worse than the trained classification results, which indicated that there was a certain degree of over-fit in our model training, which is often affected by the number of features and the complexity of the algorithm. Overfitting makes the model more susceptible to noise and reduces the accuracy of the predictions, but it is also unavoidable. However, in our 10-fold validation results, the model also showed a good ROC–AUC index, so although the Bayesian model we developed may have some overfitting, it is still considered to have good distinguishability.

**Figure 5:**
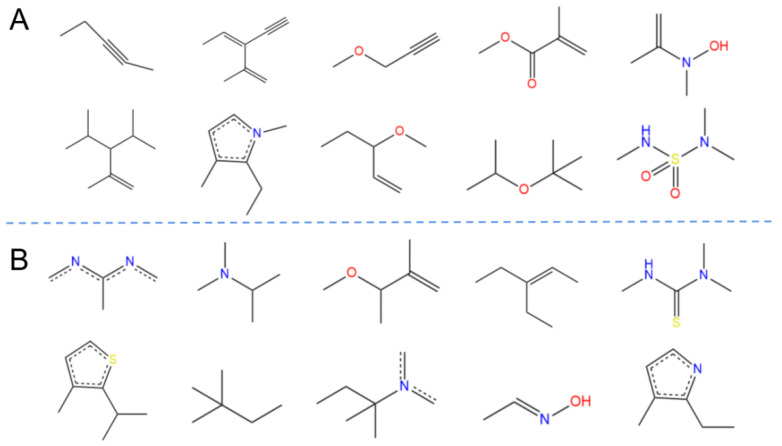
Favorable/unfavorable active fragments calculated based on ECFP_6: (**A**) top 10 good fingerprints (GF1–GF10) calculated based on ECFP_6 fingerprints; (**B**) top 10 bad fingerprints (BF1–BF10) calculated based on ECFP_6 fingerprints.

We calculated the high-frequency good/bad feature fragments (GF/BF) based on ECFP_6 fingerprinting for all compounds classified as active (171) and inactive (279). Because the frequency of molecular fingerprints of all 171 compounds classified as “active” by the Bayesian classifier was considered in the calculation of the favorable fragments, the fragments in Figure 5 represent only the statistical results based on a large amount of data for the structure–activity relationship study of potential ALOX15 inhibitors. For the favorable fragments GF1–GF10 calculated via molecular fingerprinting, a larger proportion of fragments contained ether, carboxyl, or ester bonds.

### 2.5. Refined Molecular Docking Analysis

**Figure 6:**
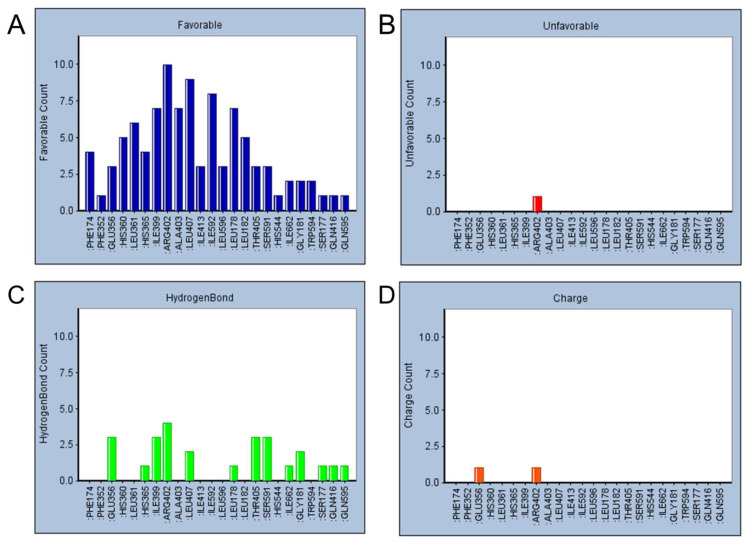
Heat map of favorable interaction types and residue statistics for the ten available active compounds: (**A**) total counts of favorable interactions formed between the top 10 best available compounds and target residues; (**B**) counts of hydrophobic interactions formed between the top 10 best available compounds and target residues; (**C**) counts of hydrogen bonds formed between the top 10 best available compounds and target residues; (**D**) counts of electrostatic charge interactions formed between the top 10 best available compounds and target residues.

In the previous high-throughput docking and classifier predictions, 10 compounds that were predicted to have a good effect were selected. To analyze how these compounds interact with the targets, compound–target precision docking was conducted using the CDOCKER module. First, the docking residues and types of action of the 10 candidate compounds were counted. As displayed in Figure 6, six residues, namely Leu178, Ile399, Arg402, Ala403, Leu407, and Ile592, produced the most favorable interactions with the ligands. Most of these interactions were hydrophobic interactions. The study by Meng et al. in 2018 revealed Phe174, His360, Ile399, Arg402, Ala403, Leu407, and Leu596 as the key ALOX15 residues, consistent with the statistical results. In 2020, Cruz et al. revealed that residue 596 plays a key role in maintaining the structural stability of ALOX15 [27].

**Figure 7:**
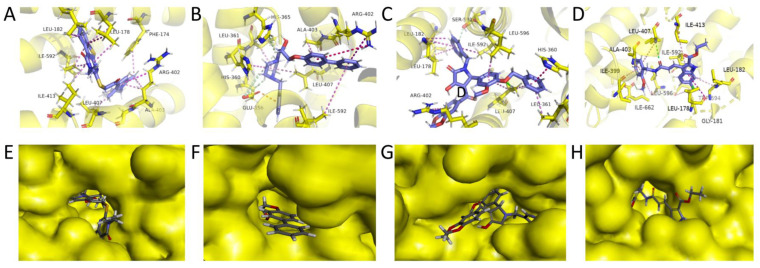
Schematic 3D interactions of the best compounds C1–C3 with the target: (**A**) schematic of the interaction of compound C1 with residues within the target-binding pocket of ALOX15; (**B**) schematic of the interaction of compound C2 with residues within the target-binding pocket; (**C**) schematic of the interaction of compound C3 with residues within the target-binding pocket; (**D**) schematic of the interaction of compound i472 with residues within the target-binding pocket; (**E**) mosaic binding of compound C1 within the target-binding pocket; (**F**) mosaic binding of compound C2 within the target-binding pocket; (**G**) mosaic binding of compound C3 within the target-binding pocket binding; (**H**) mosaic binding of compound i472 in the target-binding pocket.

Guo et al. obtained a novel ALOX15 inhibitory compound “i472” as a control [28] by using the CDOCKER docking fraction (including both CDOCKER ENERGY and CDOCKER INTERACTION ENERGY). The three most promising compounds (the first three compounds presented in Table 7) were identified in terms of their CDOCKER ENERGY (which is based on the intermolecular interaction energy, whereas in the CDOCKER INTERACTION ENERGY, the intramolecular energy is considered), type of interaction, and their interactions with key residues. These compounds were labeled C1–C3. The three-dimensional (3D) docking schematics are displayed in Figure 7A–D.

Considering the van der Waals forces between the ligands and the residues, all three of the selected compounds interact with the key residue 596, which revealed the excellent target-binding potential of the selected compounds.

**Figure 8:**
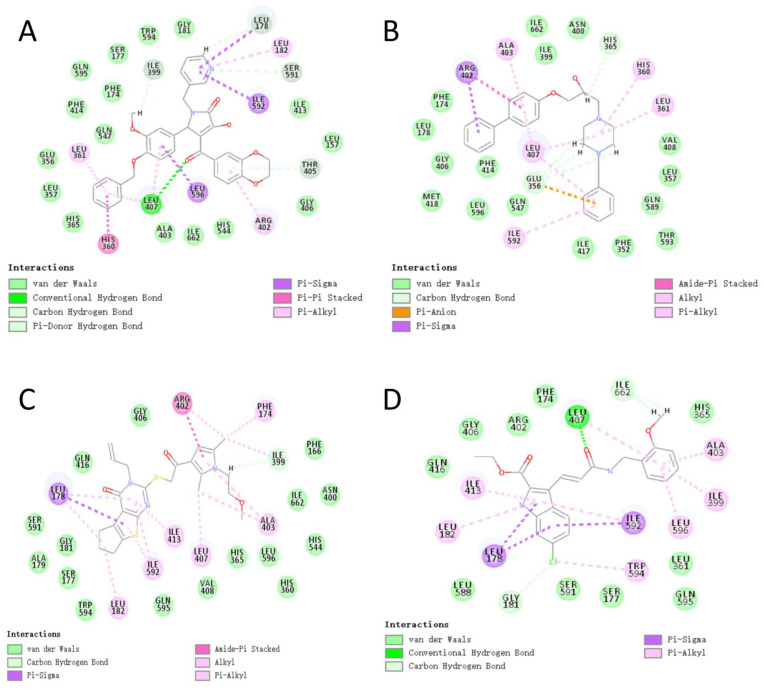
Two-dimensional (2D) diagrams of the interactions of the three best compounds and the control compound i472 with the target: (**A**) schematic of the plane of interaction of compound C1 with the target; (**B**) schematic of the plane of interaction of compound C2 with the target; (**C**) schematic of the plane of interaction of compound C3 with the target; (**D**) schematic of the plane of interaction of the control compound i472 with the target.

For the best scoring compound C1, as displayed in Figure 8A, the target–ligand interactions were dominated by Pi interactions. Leu178, Leu182, His360, Leu361, Arg402, Leu407, Ile592, and Leu596 were among the key Pi-type residues. This result is consistent with those reported by Meng et al. According to the 3D structure of the complex, ligand C1 was essentially fully embedded in the target active residue pocket, resulting in the formation of more van der Waals interactions with the receptor, which enhanced its target binding.

For compound C2, the formation of Pi bond interactions between its residues, Glu356, His360, Leu361, Arg402, Ala403, Leu407, and Ile592, and the targets, including Pi-anion, Pi-sigma, and Pi-alkyl types, account for the compound’s hexameric ring structure (Figure 8B). Furthermore, Glu356 formed a strong carbon–hydrogen bond with the hydrogen atom on the diazohexa ring of the small molecule while forming a Pi-anion interaction with the ligand, contributing to the ligand–acceptor interaction. Taking the carbon atom connected to the hydroxyl group on the backbone of compound 2 as the stereogenic center, the R/S configuration of the two enantiomers of each other can be observed. We only considered the R configuration of compound 2 during the above screening. Therefore, we additionally docked the chiral S configuration of compound 2 to the target. However, during docking, the S configuration fails to successfully bind to the protein structure of ALOX15. Therefore, we believe that the R conformation of compound 2 is promising.

The interaction of compound C3 with the target is displayed in Figure 8C. Numerous Pi interactions can contribute to the ligand–receptor interaction. Similarly, the key residues Phe174, Leu178, Arg402, Ala403, Leu407, and Ile592 formed Pi interactions with the ligand, which confirmed the reliability of the docking analysis.

The control compound i472 formed Pi-alkyl interactions with the known target key residues Ala403 and Leu596 (Figure 8D). Overall, the compound exhibited similarities to our three candidate compounds in the type and number of target interactions. However, i472 formed fewer van der Waals interactions with protein residues. This result could be attributed to the ability of the three alternative compounds to access the active residue pocket more deeply than i472 (Figure 7E–H). The fine-grained interaction force analysis allowed the identification of three candidate compounds with an ALOX15 targeting ability comparable to that of compound i472.

Comparing previous favorable fragments obtained from molecular fingerprinting calculations, the favorable fragments of the top 10 compounds were identified and are highlighted in light red in the molecular structure diagrams in Table 7. In the top 10 compounds and the control compound i472, the favorable fragment GF4 containing an ester bond, a carbon–carbon double-bond structure, and the nitrogen-containing five-membered ring structure GF7 was observed, whereas for our three selected alternative compounds C1–C3, the favorable fragment 7 (GF7) was observed in the C1 and C3 structures. No predicted favorable fragment was observed in compound C2. Considering that these three compounds have the highest docking scores and target-binding ability, GF7, a nitrogen-containing five-membered ring structure can be assumed to be of some significance for the identification of ALOX15 inhibitors. However, when the hydrogen atom that linked to the nitrogen atom on the five-membered ring of the favorable fragment 7 was replaced by other groups (bad fragment 10), the activity was adversely affected. This result can provide guidelines for the conformational studies of ALOX15 inhibitors.

### 2.6. ADMET Property Prediction

**Figure 9:**
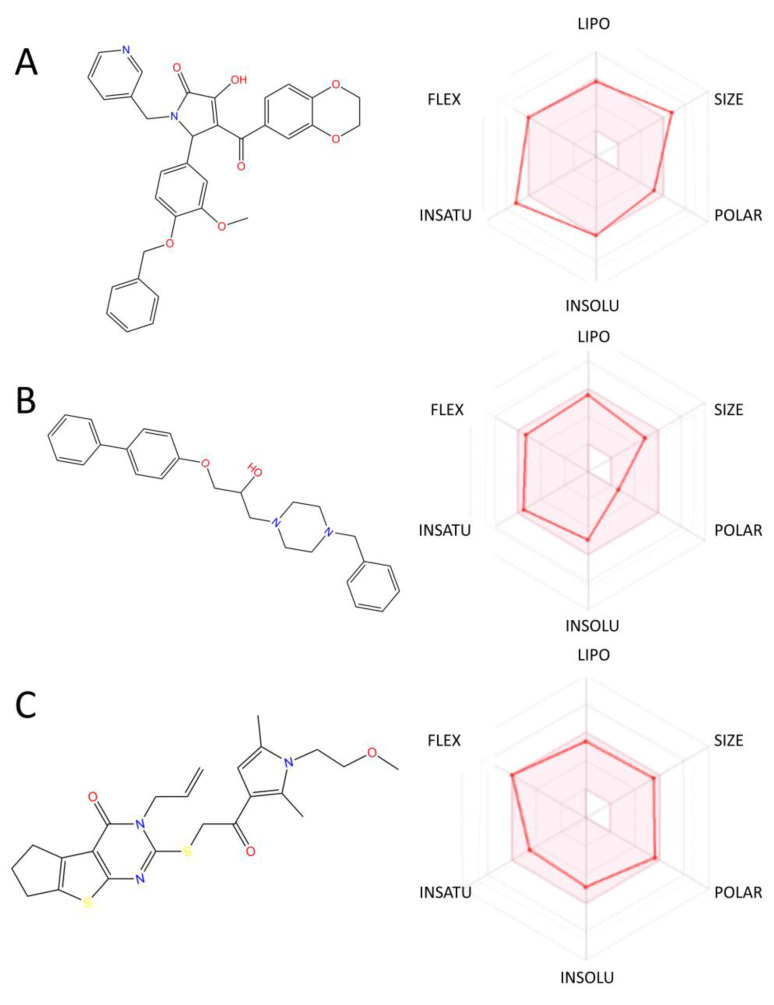
Distribution of the hexagonal range of ADME properties for candidates C1–C3: (**A**) distribution of ADME properties for compound C1; (**B**) distribution of ADME properties for compound C2; (**C**) distribution of ADME properties for compound C3.

SwissADME was used to predict the absorption, in vivo distribution, metabolism, excretion, and toxicity properties of the three compounds that were identified as ALOX15 targets. First, the in vivo availability of these compounds, including the water solubility, lipid solubility and BBB penetration, gastrointestinal absorption (GI absorption), and inhibition of P-gp drug metabolism proteins, was investigated. The corresponding property indices for the compounds tested are presented in Figure 9 and Table 8.

The LogSw value represents the water solubility coefficient of the drug. The lower this value than *−*4.0, the less soluble the compound is in water; LogSw <−6.0 reveals that the compound is barely soluble in water, as detailed in Table 8. The predicted LogSw value for C1 was less than −6.0, whereas the remaining two molecules were poorly soluble in water. This result could be related to the molecular structure containing fewer ionizable groups. Furthermore, the compounds formed fewer hydrogen bonding interactions with the target protein and more Pi-polar interactions, which contributed to their low water solubility.

The LogP value represents the oil–water partition coefficient of a substance and is the logarithmic value of the ratio of the partition coefficients of a substance in n-octanol and water. Thus, a high LogP value indicates strong lipophilicity of the compound, whereas a low LogP value indicates weak hydrophilicity of the compound. C1–C3 showed similar lipophilicity levels, with consensus Log Po/w values of 3.66, 3.74, and 4.22, respectively, indicating that these alternative compounds have a partition ratio close to 4:1 in the organic and aqueous phases and are soluble in organic oil solvents. This result is consistent with their predicted low water solubility.

P-glycoprotein 1, also known as permeable glycoprotein (P-gp), pumps foreign drugs out of cells in an ATP-dependent manner with broad substrate specificity [29]. When a drug is available as a substrate for P-gp, its in vivo availability is reduced. Compounds C1 and C3 are predicted to be substrates for P-gp; however, their predicted bioavailability values are not reduced compared with that of C2, which we assume to be related to the low water solubility of these compounds. The blood–brain barrier (BBB) permeability of small molecules depends on their lipid solubility, molecular complexity, polar surface area, and whether they are P-gp substrates. Crossing the BBB is inherently difficult for hydrophilic large-molecule drugs, whereas some lipophilic suitable molecular weight drugs can cross the BBB but are easily transported by efflux pumps, such as glycoproteins (Pgp), on the BBB. The predicted results revealed that only compound C3 had BBB permeability.

The Lipinski, Ghose and Veber, Egar, and Muegge rules were used in the module for the evaluation of molecules. The Lipinski rules for the evaluation of druggability are widely used [30] and include five main principles that should be satisfied if a compound exhibits formulation properties: a molecular weight of less than 500, hydrogen bond donor number of less than 5, hydrogen bond acceptor number of less than 10, lipid-water partition coefficient of less than 5, and rotatable bond number that does not exceed 10. As shown in the results displayed in Table 8, all compounds satisfied the drug-formulating rules, which revealed excellent drug properties, with the exception of compound C1, which violated one of the Lipinski rules because its molecular mass was slightly higher than 500.

Finally, the potential toxicity of these candidate compounds was assessed using the quantitative structure–toxicity relationship model of the TOPKAT module. The results are presented in Table 9 and all three compounds were predicted to be non-AMES mutagenic. In the FDA-standard mouse model, both alternative compounds were predicted to be non-carcinogenic, with the exception of compound 3al, which had a single possible carcinogenic toxicity to female mice.

### 2.7. Molecular Dynamics Simulation

**Figure 10:**
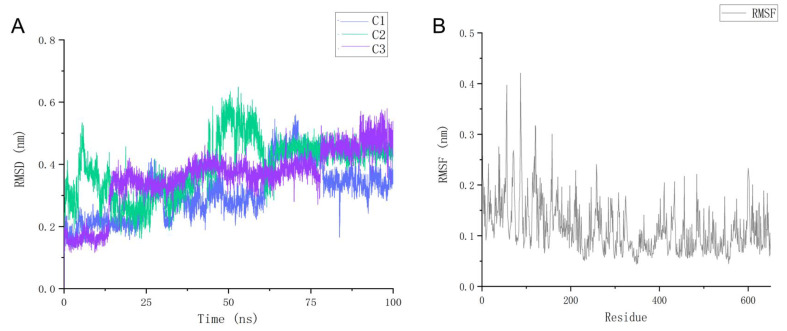
Results of molecular dynamics calculations performed on the candidate compound–protein receptor complexes: (**A**) the changes in conformational RMSD values for the three candidate ligands during the 100 ns kinetic simulation; (**B**) the changes in conformational RMSF values for the receptor protein residues over 100 ns.

To confirm whether the binding of the candidate compounds to the target is sustained and stable, we performed molecular dynamics simulations for 100 ns on three ligand–receptor complexes and obtained the RMSD values of the ligands and RMSF data for the protein residues. As displayed in Figure 10A, all three candidate ligands exhibited stable conformational RMSD fluctuations over the 100 ns duration. C1 exhibited a slight increase in RMSD values throughout the 100 ns period but remained generally flat. C2 showed non-significant differences in RMSD values at the beginning and end of the MD, but clear conformational peaks were observed at the 4–12 and 47–53 ns time periods. Subsequently, the RMSD values rapidly returned to their initial levels without large fluctuations, which still indicates that its stable ALOX15 binding properties mimic the in vivo solvent environment. For compound C3, its RMSD values maintained a steady upward trend throughout the MD process but did not exhibit significant fluctuations. By the end of 100 ns, the final RMSD values for the three candidate compounds were similar (C1: 0.382 nm; C2: 0.426 nm; C3: 0.424 nm), which indicated that all three complex systems reached a similar steady state within a short time.

The RMSF of a protein residue represents the root mean square displacement of the residue in the protein conformation; that is, its flexibility. As displayed in Figure 10B, the RMSF values for the proteins ranged from 0.03 to 0.44 nm. The front approximately 220 residues exhibited high RMSF values, which indicated their high flexibility in salt solutions. Residues further back in the sequence (which also host the protein active site) exhibited low RMSF values throughout because of their more stable binding to the ligand. For the key active binding residues of the target, Arg402, Leu407, and Leu596 (represented here by the first three favorably acting ranked residues in the molecular docking results section), their RMSF values of 0.08, 0.06, and 0.06 nm were all low, indicating that they form stable interactions with the candidate compound molecules. This result confirmed the previous molecular docking results. The RMSD and RMSF data revealed that the three candidate compounds exhibited stable binding properties to their targets during the 100 ns aqueous environmental kinetic simulations.

## 3. Discussion

Ferroptosis is a critical cell-regulated death pathway that causes irreversible damage to body tissues. The peroxidation of unsaturated lipids (PUFAS) is a key mechanism for the induction of cellular ferroptosis. ALOX15 is a non-hemoglobin-dependent dioxygenase that directly oxidizes PUFAs in biological membranes and leads to the accumulation of a series of intracellular peroxidized lipids and ferroptosis [14]. Thus, ALOX15 has become a popular target in the study of many diseases, including tumors and degenerative diseases. The role played by ALOX15 in programmed cell death and abnormal metabolism has long been recognized. The role of ALOX family enzymes in ferroptosis was first reported in the study by Yang et al. in 2016 [11], which promoted research interest in ALOX15 enzyme inhibitors. During 2016–2019, numerous ALOX15 inhibitors were reported. The most representative model was the compound i472, designed and synthesized by Guo et al. based on the structure–activity relationship study approach [28], which was protective against macrophage death induced by external toxic factors. Golovanov et al. in February 2022 revealed that a combination of conventional chemical design–synthesis techniques and molecular dynamics simulations were applied to confirm the ALOX15 inhibition capacity of the N-substituted 5-(1 H-Indol-2-yl)-2-methoxyanilines family of compounds [17]. In March of the same year, Aghasizadeh et al. proved the excellent inhibitory effect of 8-Geranyloxycarbostyril on ALOX15 in human PCa cells through cellular assays and in vitro mouse models [31]. Although many ALOX15 inhibitory compounds have been reported for experimental use, the true ALOX15 molecules that can be used for clinical treatment are yet to be discovered, and options and possibilities should be explored. With the rapid development of computer technology, CADD has been widely used to rapidly screen out molecules with excellent target-binding potential from a large number of compounds, which results in significant cost savings for numerous experimental consumables. A large-scale virtual screening based on molecular structure was conducted.

Experimentally extracted proteins can be subjected to enzymatic digestion and cryo-electron microscopy to obtain amino acid sequences and 3D structures that can be used in virtual research processes. However, for many proteins used as targets for drug research, such as ALOX15, limited accessible structure files have been developed for proteins of Homo sapiens origin, and the experimental dataset collected by the BLAST sequence database provides a solution to this problem. Given that homology modeling studies are yet to be conducted for human-derived ALOX15 proteins, this operation was performed using numerous computational tools. The structural models proved to be highly reliable and could provide the basis for subsequent CADD studies on this target.

Machine learning methods have also been increasingly applied in the drug discovery process, such as the differentiation of active and inactive molecules and the prediction of drug-like and non-drug-like compounds. Bayesian algorithms tend to exhibit excellently recognition under dichotomous scenarios. Based on a collection of high/low-activity ALOX15 inhibitor molecules from previous studies, the Bayesian classifier was constructed to classify molecules from commercial compounds after initial structural screening. The PCA of the molecular descriptors revealed that the dataset used to construct the classifier model was broad and sufficiently dispersed to provide a basis for the reliability of the constructed model. The results of the test set and the 10-fold validation showed that the Bayesian model allows for the excellent discrimination of inactive molecules. However, this also resulted in a high false positive rate, i.e., the compounds we classified as “active” were likely to contain compounds that did not actually have target inhibitory activity. Given this situation, we went a step further and performed a precise docking analysis of the selected compounds to confirm whether the molecules identified by the classification model actually bind to the target and how well they interact with the target. On the other hand, for the ECFP fingerprints used to characterize the structure of the compounds, the fingerprint radius is also likely to have a significant impact on the classification performance of the model, as it relates to how large a molecular fragment the classification learning model will use as a training reference. We also present the top 10 most frequent fingerprints of all molecules classified as ‘active’ and ‘inactive’. For the compounds predicted to be ‘active’, structures with unsaturated ether and ester bonds were the most frequent fingerprints. The fragments containing nitrogenous five-membered rings also appear to be critical when considered in conjunction with the structures of the 10 compounds we selected as the best predictors. Overall, although we did not consider the classification efficacy of more machine learning models, the performance of the Bayesian classifier on the active training set was adequate for our screening.

In a precise molecular docking analysis, the CDOCKER ENERGY and CDOCKER INTERACTION ENERGY values were calculated, i.e., both the internal energy of the ligand molecule and the external target-binding energy were taken into account. The final three most promising compounds identified were very similar to the positive control molecule and to the key binding residues of ALOX15 in terms of the type of target-binding action. This highlights their potential as target inhibitors. Molecular dynamics studies of the ligand–target complexes confirmed the results in terms of fine docking; although the compounds showed “peak-like” conformational fluctuations over several small time intervals, their RMSD values eventually stabilized in similar intervals, confirming the stable interaction between the ligand and protein. Finally, we predicted the drug-likeness of the three candidate compounds C1–C3 using the ADMET prediction program, which showed excellent druggability for all candidates, as well as low teratogenicity and low carcinogenicity in mice. Although C1–C3 showed excellent dummy predictive properties, their true ALOX15 inhibitory activity has to be verified by further experimental data. Overall, these three compounds showed excellent target-binding potential and druggability and could be used as a reference for further development of ALOX15 inhibitors.

## 4. Materials and Methods

### 4.1. Protein Homology Modeling

For protein-based virtual screening processes, a known target protein structure is mandatory. As no existing human-derived ALOX15 protein structures were retrieved on the PDB database, we first searched to obtain the rabbit-derived reticulocyte ALOX15 protein sequence (PDB ID: 2P0M). By searching the nr_clustered experimental database from the UniProt KB [32] database, we performed a balstp algorithm on the rabbit ALOX15 sequence to obtain the human-derived ALOX15 with protein sequences with high similarity. The search parameter was set to PDB_nr95, i.e., the templates were searched in an information database with no redundancy at 95% sequence homology. The searched templates were sorted by the E value size, with lower E values indicating the higher reliability of the sequence alignment [33].

Subsequently, the human-derived ALOX15 protein model was constructed using the MODELLER module [34] on the Discovery Studio 4.5 platform. The accuracy of the model was calculated by calculating the model-verified score.

For pre-processing of the homologous model, we used the Prepare Ligand module of the DS platform for the energy and structural optimization of the protein model. The loops structure of the protein was defined using the SEQRES algorithm, while a CHARMM force field [35] was applied to the structure for energy minimization and protonation; the Dielectric constant was set to 10, while the pH for protonation was set to 7.4 in order to keep the protonation acid–base stable.

### 4.2. Protein Model Validation

The quality and spatial distributions of the residues of the constructed protein models were evaluated using the DS platform’s MODELLER and Profile-3D scoring functions and Ramachandran plots. For each model evaluated, an expected high score, expected low score, and verify score were assigned. When the verify score is higher than the expected low score, the reliability of the model is guaranteed; the closer the verify score is to the expected high score, the higher the refinement (quality) of the model. The Ramachandran plot [36] is used to evaluate the spatial distribution of two adjacent peptide unit backbones by the minimum non-bonded atomic contact distance. In general, a reasonable protein model should have no more than 5% of the total number of residues located in the irrational region.

### 4.3. Structure-Based High-Throughput Molecular Screening

We selected 210,070 molecules from the SPECS commercial compound library and performed structure-based virtual screening based on homologous modelled proteins with the help of the LibDock [37] module of the Discovery Studio platform. The protein models were pre-corrected for structure by the Macromolecules module. The active site residues (Thr412, Arg415, Val420, Thr429, Ile602, and Trp606) were identified by searching the protein structure literature [38] with docking radius sphere 3D coordinates of −55.173933, 169.711977, and 32.549350. In the LibDock module, 100 spatial hotspots are randomly generated in a docking radius sphere, and compound conformations are rapidly matched to each hotspot and analyzed for good interactions with protein structures. Molecules contained within the compound library were pre-hydrogenated and charge-balanced by the Prepare Ligand module and all possible docking conformations were generated to ensure diversity of docking poses and accuracy of results.

### 4.4. Molecular Descriptor Calculation and Principal Component Analysis

Here, 5360 ALOX15 inhibitory compounds from the CHEMBL database were collected from the ExCAPE database (https://solr.ideaconsult.net/search/excape/, accessed on 13 August 2022) [39]. The compounds were first subjected to energy minimization and valence bond repair by applying the Prepare Molecule module in Discovery Studio. Subsequently, 55 molecular physicochemical property descriptors were calculated for the ALOX15 inhibitors collected from the ExCAPE database using the Calculate Molecular Properties function. In order to avoid duplicate intersections between the SPECS screening set and the ALOX15 inhibitor molecules, the DS Find Similar function was used to search for possible similar molecules to ensure that there were no duplicate components between the SPECS dataset and the ALOX15 inhibitor set. The module was used to calculate the principal components of these descriptors in order to perform data dimensionality reductions. The Pearson correlation analysis method [40] was used to calculate the activity correlation coefficient for each descriptor, setting the minimum variance explained to 0.9, i.e., excluding descriptors with activity correlation coefficients below 0.1 and retaining key descriptors with coefficients above 0.9. The minimum number of components was 3. The OPS analysis method was applied to the calculation of principal components. The correlation coefficients preceding each descriptor in the PC equation calculated from the principal component analysis reflect the magnitude of their contribution to the principal component. The descriptor with the largest contribution in aggregate was selected as the base property for constructing the machine learning classifier. The ECFP_2 fingerprint was also calculated for the dataset compounds to calculate the Tanimoto distance of the features [41]. Extended connectivity fingerprinting (ECFP_n) is a class of 2D ring fingerprint tool based on a variant of Morgan’s algorithm that captures atomic information based on daylight atomic invariant rules [42]. It sets radius “n” as the number of iterations to calculate the atomic environment identifier. Compared to molecular physical and chemical property descriptors, ECFP fingerprints are more focused on the chemical backbone information of molecules. Thus, it is possible to provide machine learning with structural reference information for individual training set molecules. The fingerprint features were tracked during the computation and the minimum sample of each variable was set to SqrtEstimate. in the process of scaling the data, while all features being computed were averaged and centered.

### 4.5. Machine Learning Classifier Construction

The 5360 ExCAPE compounds were pre-flagged as active and inactive (activity flag) based on PXC50 values: compounds with PXC50 values >5 were considered active in the definition given by the database itself, equating to IC50/EC50 dose–response values ≤10 μM. The others were inactive (1835 active compounds and 3525 inactive compounds, an active/inactive ratio close to 1:2). The collected active molecule data were converted to the nominal form, normalized by the unsupervised metric filter in WEKA software [43], and saved in the arff format for use in the subsequent classifier construction.

The NB classifier is an efficient machine learning algorithm based on Bayes’ theorem and is often used to classify training sets with a large number of samples because of its immunity to random noise during training [44]. *P*(*A*) in Equation (1) represents the prior probability of independent event A occurring; *P*(*B*) is the prior probability of independent event B occurring; *P*(*B*|*A*) is the posterior probability of event *B* occurring when event *A* occurs; *P*(*A*|*B*) is the posterior probability of event *A* occurring if independent event *B* is known to occur. Thus, the Bayesian algorithm is essentially a modification of the prior probability of the occurrence of independent events, which after a suitable number of validation modifications ultimately gives the best binary validation model. In several earlier examples of drug research, Bayesian models have demonstrated excellent discriminatory power in dichotomous cases [45], which is why we decided to choose the NB model.

We used the key molecular descriptors calculated from the principal component analysis to assist in the construction of the model. At the same time, a series of ECFP fingerprints (ECFP_0, ECFP_2, ECFP_4, ECFP_6, ECFP_8) were included to ensure that the structural features of the molecules were fully considered in the construction of the classification model. The classifier construction method was then tested using a 10-fold validation method. This method randomly divides the original dataset into 10 equally sized subsets, one of which is selected in turn as the validation set, until each subset is individually selected for validation. The 10-fold validation method has proven to be the most effective algorithm testing procedure in a large number of dataset testing examples [46]. The true positive rate, false positive rate, area under the ROC curve, precision, recall, F-measure, and Matthews correlation coefficient (MCC) values were used as metrics to evaluate the model.

The ROC curve (receiver operating characteristic curve) is often used to characterize the ability of the model to discriminate between positive and negative samples. It takes the true positive rate (TPR, also sensitivity) as the Y-axis and the false positive rate (FPR, also 1-specificity) as the X-axis, and gives a coordinate point representing the optimal classification threshold in the coordinate species; the smaller the distance between this coordinate point and the top left corner of the curve axis, the closer the area under the curve (AUC) value will be to 1, and the better the model’s TPR (sensitivity) and specificity, which are two different dimensions of the ROC curve used to characterize the recognition ability of the model. The latter represents the ratio of inactive compounds correctly classified by the model for all actual inactive compounds, representing the classifier’s ability to identify negative examples.

The recall value, also known as the sensitivity, represents the percentage of true positives that are correctly classified (Equation (2)), while the specificity value, in contrast to the recall value, represents the percentage of true negatives that are correctly classified (Equation (3)). The F-measure, also known as the F-score, is the weighted summed average of the precision and recall values (Equation (5)). When the equation parameter is set to 1 (α = 1), the resulting F-measure is also known as the F-1 score, which gives the same weight to the precision and recall values and is often used to evaluate the strength of a model’s classification; higher F-1 values tend to indicate higher model validity.

The MCC (Matthews correlation coefficient) is a Pearson product moment correlation coefficient (Equation (6)) based on a weighting matrix that takes into account the true positive, true negative, false positive, and false negative rates of the classification, and is often used as a measure of the classification performance of a dichotomous model. The metric has a wide range of applicability and is also good for assessing samples with uneven distribution [47]. The value range is [−1, 1], with values closer to 1 indicating that the classifier is more accurate, values of 0 indicating that the classifier’s predicted results are worse than those predicted by random classification, and values closer to −1 suggesting that the predicted classification results deviate more from the actual classification.

(1) $$P\left(A|B\right)=\frac{P(B|A)*P\left(A\right)}{P\left(B\right)}$$

(2) $$Sensitivity=Recall=\frac{TP}{\left(TP+FN\right)}$$

(3) $$\;Specificity=\frac{TN}{\left(FP+TN\right)}$$

(4) $$Precision=\frac{TP}{\left(TP+FP\right)}$$

(5) $$F-Measure=\frac{\left(1+\alpha 2\right)*precision*recall}{precision+recall}$$

(6) $$MCC=\frac{TP*TN-FP*FN}{\surd \left(TP+FP\right)\left(TP+FN\right)\left(TN+FP\right)\left(TN+FN\right)}$$

### 4.6. High Accuracy Molecular Docking

To further explore the interactions between molecules selected by the high-throughput structure screen and ML classifier and target residues, we applied the CDOCKER module [48] of the DS platform to perform a docking analysis of alternative inhibitor molecules. Similar to the high-throughput structural screen we ran previously, the protein models obtained from the homology modelling were used as docking targets. During the fine docking, the CHARMM force field was applied and the program generated 10 random body images for each docked molecule through short time duration (1000 steps) molecular dynamics simulations. The maximum temperature of the kinetic process was 1000 K. During this process, the electrostatic interactions formed between the small molecule and the target were also taken into account. Meanwhile, the orientation for refining was set to 10 to ensure the diversity of the docking conformations, while the maximum number of bad orientations was set to 800 and the Momary–Rone method was used as the ligand partial charge method. In the final step of the ligand minimization process, full potential was supplied, but no gradient tolerance of minimization was allowed. The grid extension was set to 8.0.

### 4.7. ADMET Property Prediction

In addition to the ability of the small molecules to bind to their targets, stable in vivo metabolic properties and low compound toxicity are also important indicators of a compound’s potential to become a drug. Therefore, we need to further determine the ADMET (i.e.,) properties of compounds in order to exclude molecules with poor drug-forming potential from the alternative compounds. Conventional methods for the evaluation of ADMET properties are performed with the aid of cellular or animal experiments, but such methods do not meet efficiency and cost optimization considerations for the large number of compounds to be tested [49]. In the present study, we used the SwissADME (http://www.swissadme.ch/, accessed on 10 September 2022) [50] online tool developed by Antoine et al. to predict the ADMET properties of the compounds to be determined. The website predicts the drug-generating properties by importing compound smile files, including the physicochemical parameters, pharmacokinetic characteristics, and drug-likeness. Compounds identified after the previous steps are generated using the Openbabel software with the corresponding smile files and imported into SwissADME to generate the drug-generating properties of the compounds. Additionally, the teratogenicity, carcinogenicity, and hepatotoxicity of the alternative compounds were predicted using the TOPKAT toxicity prediction module [51] from the DS platform.

### 4.8. Molecular Kinetic Simulations

A molecular dynamics simulation (MD) is a molecular simulation technique based on Newtonian mechanics theory that has been applied to the monitoring of thermodynamic reactions within a single system. MD methods are widely used in computer-assisted drug development studies to examine whether a pre-drug compound can bind stably to a specified target. To examine the target-binding ability of the three candidate molecules identified by the above process, we ran a 100 ns aqueous solution environmental kinetic simulation based on the ligand–receptor complexes previously obtained via accurate docking using the GROMACS 2019.1 (sourced by Mark Abraham et al. from Uppsala University, Stockholm University and the Royal Institute of Technology, Sweden) software package [52]. First, the complex system was constructed. The PDB format files of the ALOX15 homologous protein model and candidate ligand molecules were obtained via transformation using the Openbabel tool [53]. The AMBER99SB-ILDN force field was used to construct the topological system of the protein. For the ligands, we submitted a GAFF force field-based topology scheme from the ACPYPE online server [54] (https://www.bio2byte.be/acpype/, access on 19 September 2022) to obtain the force field parameters of the compounds. A cubic box with a radius of 1.2 nm was constructed and centered on the integrated resulting complex system, to which the SPC216 water model was added to simulate an aqueous solution environment. To ensure the total charge neutrality of the simulated system, corresponding amounts of sodium and chloride ions were added to the system to replace the water molecules. Periodic boundary conditions (PBC) were applied to the three directions of the spatial coordinates of the complex system.

The energy minimization of the system was carried out in 50,000 steps at a simulated temperature of 300 K, provided that the whole complex system had been prepared. After correction for positional constraints, equilibrium for the receptor, ligand, and solvent is achieved through a constant temperature and volume (NVT) and a constant temperature and pressure (NPT) conditioning process, respectively. Finally, MD simulations of 100 ns in duration were run.

## 5. Conclusions

In this study, we identified three compounds with excellent ALOX15 target-binding potential through computer-assisted screening processes. In the screening phase, a machine learning classifier constructed based on the Bayes method was combined with a conventional structure-based, high-throughput screening approach to accurately select suitable molecules. To validate the drug-forming potential of these selected molecules, a fine-grained docking analysis was applied to the process and the three compounds had a target-binding pattern similar to that of the positive control molecule. The stability of the binding was also confirmed by MD simulations. The ADMET property predictions indicated that the drug-forming potential of the compounds was desirable. The three newly identified ALOX15 inhibitory potential compounds in this study exhibit the potential to inhibit the development of cellular ferroptosis and provide a reference for the treatment of tissue-damaging diseases.

## Figures and Tables

**Table 1:** Verified score for the homologous protein model M0018.

Name	Verify Score	Verify Expected High Score	Verify Expected Low Score
M0018	278.82	303.15	136.417

**Table 2:** Alignment cluster root mean square deviation (RMSD) values for 2P0M and M0018 (unit: Angstrom).

Protein	2P0M	M0018
2P0M	--	1.1630
M0018	1.2990	--

**Table 3:** Training metrics for the best naive Bayesian (NB) model.

Class	Precision	Recall	F-Measure	AUC	MCC
Active	0.847	0.925	0.884	0.895	0.838
Inactive	0.958	0.913	0.935		
Weighted Avg	0.903	0.919	0.910		

**Table 4:** Training activity/inactivity confusion matrix for the best NB model.

Classification	Predicted Active	Predicted Inactive
Active	1697	138
Inactive	305	3220

**Table 5:** Parameters of the ten-fold validation results for the NB model.

Class	Precision	Recall	F-Measure	AUC	MCC
Active	0.782	0.855	0.817	0.745	0.478
Inactive	0.920	0.876	0.897		
Weighted Avg	0.851	0.866	0.857		

**Table 6:** Active/inactive confusion matrix for ten-fold validation of the NB model.

Classification	Predicted Active	Predicted Inactive
Active	1569	266
Inactive	437	3088

**Table 7:** Chemical names, molecular structures, and docking fractions of candidate compounds 1–10.

Index	Name	Structure	LibDock Score	CDOCKER Energy	CDOCKER Interaction Energy
1	5-[4-(benzyloxy)-3-methoxyphenyl]-4-(2,3-dihydro-1,4-benzodioxin-6-ylcarbonyl)-3-hydroxy-1-(3-pyridinylmethyl)-1,5-dihydro-2H-pyrrol-2-one **(C1)**	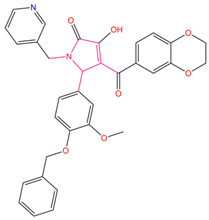	156.52	−43.897	−36.283
2	1-(4-benzylpiperazin-1-yl)-3-([1,1′-biphenyl]-4-yloxy)propan-2-ol **(R)** **(C2)**	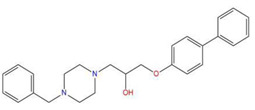	129.364	−32.940	−25.541
3	3-allyl-2-({2-[1-(2-methoxyethyl)-2,5-dimethyl-1H-pyrrol-3-yl]-2-oxoethyl}sulfanyl)-3,5,6,7-tetrahydro-4H-cyclopenta [4,5]thieno [2,3-d]pyrimidin-4-one **(C3)**	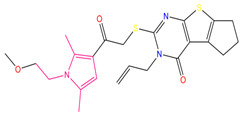	133.492	−35.653	−29.243
4	1-{7-acetyl-9-[4-(octyloxy)benzylidene]-9H-fluoren-2-yl}ethanone	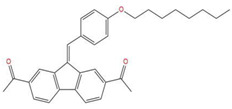	127.735	−34.728	−20.403
5	2-chloro-*N*-[2-({2-oxo-2-[(1-phenylethyl)amino]ethyl}sulfanyl)-1,3-benzothiazol-6-yl]benzamide	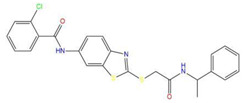	118.925	−23.495	−15.965
6	4-bromo-*N*-[2-(2-cyclooctylidenehydrazino)-2-oxoethyl]-*N*-(4-ethoxyphenyl)benzenesulfonamide	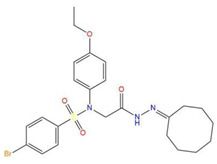	121.455	−25.582	−8.780
7	6-(4-(3-fluoro-4-methoxyphenyl)-2-{[3-(trifluoromethyl)phenyl]imino}-1,3-thiazol-3(2H)-yl)-1-hexanol	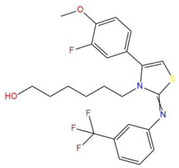	117.047	−21.340	−13.951
8	4-{(2,5-dioxo-1-phenyl-3-pyrrolidinyl)[2-(4-methoxyphenyl)ethyl]amino}-4-oxo-2-butenoic acid	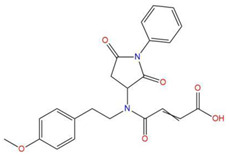	120.938	−31.054	−19.390
9	ethyl2-({4-[(dipropylamino)sulfonyl]benzoyl}amino)-5-ethyl-3-thiophenecarboxylate	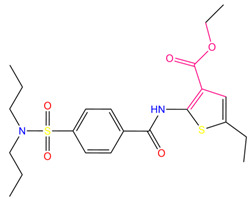	123.222	−27.384	−19.071
10	4-tert-butyl-*N*-{2-[(2-{[2-(4-chlorophenoxy)ethyl]amino}-2-oxoethyl)sulfanyl]-1,3-benzothiazol-6-yl}benzamide	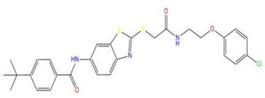	118.664	−29.755	−20.632
11	i472	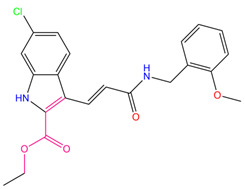	119.591	−37.382	−26.544

**Table 8:** ADME properties of candidate compounds C1–C3.

Molecule	Molecular Formula	Molecule Weight (g/mol)	Log *P*_o/w_ (iLOGP)	Log *S* (ESOL)	Solubility	BBB Permeant	Number of Hydrogen Bond Acceptor	Number of Hydrogen Bond Donor	Number of Rotatable Bond	Bioavailability Score	GI Absorption	P-gp Substrate
C1	C33H28N2O7	564.58	3.25	−6.05	Poorly soluble	No	8	1	9	0.56	High	Yes
C2	C23H27N3O3S2	457.61	4.28	−4.87	Moderately soluble	No	4	0	9	0.55	High	No
C3	C26H30N2O2	402.53	4.06	−4.92	Moderately soluble	Yes	4	1	8	0.55	High	Yes

**Table 9:** Predicted TOPKAT teratogenicity, in vivo carcinogenicity in mice, and hepatotoxicity of candidate compounds.

Molecule	Ames_Prediction	Ames_Probability	Ames_TOPKAT Score	Carcinogen_Prediction (Male/Female Mouse)	Carcinogen_Probability	Carcinogen_TOPKAT Score	Hepatotoxic_Prediction	Predicted Hepatotoxic Value
C1	Non-Mutagen	0.0764858	−18.3544	Non-Carcinogen/Non-Carcinogen	0.130807	−11.1785	false	−5.65242
C2	Non-Mutagen	0.655966	−3.35449	Non-Carcinogen/Single Carcinogen	0.211263	−4.29546	true	−2.59417
C3	Non-Mutagen	0.402773	−9.6271	Non-Carcinogen/Non-Carcinogen	0.18041	−6.31686	false	−11.614

## Data Availability

Data is contained within the article and Supplementary Material.

## Supplementary Materials

The following supporting information can be downloaded at: https://www.mdpi.com/article/10.3390/ph15111440/s1, Table S1: Top 10 protein names, source organism, PDB/BLAST accession, sequence length and E-Value evaluation values obtained from BLASTp search; Table S2: 55 molecular descriptors computed for machine learning model building; Table S3: Formulae for the composition of the top 3 key principal components; Table S4: Predicted target inhibitory activity of the Bayesian classifier for the 450 molecules with top Libdock scores.
