# Supplementary material for: Identification of Novel Arachidonic Acid 15-Lipoxygenase Inhibitors Based on the Bayesian Classifier Model and Computer-Aided High-Throughput Virtual Screening
Source: Pharmaceuticals (Basel). 2022 Nov 20;15(11):1440. doi: 10.3390/ph15111440 (PMC9695033; doi:10.3390/ph15111440)
Supplement: Supplementary file 1 [file pharmaceuticals-15-01440-s001.zip › pharmaceuticals-1987862-supplementary.pdf]

Supplementary Material

Table S1. Top 10 protein names, source organism, PDB/BLAST accession, sequence length and E-Value evaluation values obtained from BLASTp search.

Protein name	Organism	Accession	Identity(%)	Sequence Length	E-value
Arachidonate 15-lipoxygenase	*Oryctolagus cuniculus*	2P0M	100	662	0
Arachidonate 12-lipoxygenase	*Oryctolagus cuniculus*	NP_001139620.1	99.55	663	0
Arachidonate 15-lipoxygenase	*Lepus timidus*	DAZ89678.1	96.22	663	0
Polyunsaturated fatty acid lipoxygenase ALOX15	*Equus quagga*	XP_046532685.1	80.21	663	0
Arachidonate 15-lipoxygenase	*Colobus angolensis palliatus*	XP_011813735.1	81.24	662	0
Polyunsaturated fatty acid lipoxygenase ALOX15	*Homo sapiens*	NP_001131.3	81.12	662	0
Polyunsaturated fatty acid lipoxygenase ALOX15	*Ursus arctos*	XP_026376572.1	78.40	663	0
Polyunsaturated fatty acid lipoxygenase ALOX15	*Equus asinus*	XP_044601665.1	80.21	663	0
Polyunsaturated fatty acid lipoxygenase ALOX15	*Ovis aries*	XP_042111562.1	78.70	663	0
Arachidonate 15-lipoxygenase	*Condylura cristata*	XP_004684758.1	79.88	663	0

Table S2. 55 molecular descriptors computed for machine learning model building.

**Calculated Descriptors**
ALogP,LogD,Molecular_Mass,Molecular_Solubility,Molecular_Weight,QED,QED_ALERTS, QED_ALOGP,QED_AROM,QED_HBA,QED_HBD,QED_MW,QED_PSA,QED_ROTB,SAscor

e,SAscore_Complexity,SAscore_Fragments,VSA_TotalArea,HBA_Count,HBD_Count,Num_AromaticBonds,Num_AromaticRings,Num_AtomClasses,Num_Atoms,Num_Bonds,Num_ChainAssemblies,Num_Chains,Num_ComplexedFragments,Num_ExplicitAtoms,Num_ExplicitBonds,Num_Fragments,Num_H_Acceptors,Num_H_Acceptors_Lipinski,Num_H_Donors,Num_H_Donors_Lipinski,Num_Hydrogens,Num_Rings,Num_RotatableBonds,Molecular_FractionalPolarSASA,Molecular_FractionalPolarSurfaceArea,Molecular_PolarSASA,Molecular_PolarSurfaceArea,Molecular_SASA,Molecular_SAVol,Molecular_SurfaceArea, ES_Count_aaCH, ES_Count_aaN, ES_Count_aaO,ES_Count_aaNH, ES_Count_aaS, ES_Count_aaaC, ES_Count_aasN, ES_Count_aasC, ES_Count_dCH2, ES_Count_dNH, IsChiral,

Table S3. Formulae for the composition of the top 3 key principal components.

Principal Component	Composition formula
PC1	PC1 = -7.0215 + 0.078531*[ES_Count_aaCH] + 0.1513 * [ES_Count_aaN] + 0.2471 * [ES_Count_aaO] + 0.09244 * [ES_Count_aaNH] + 0.087864*[ES_Count_aaS] + 0.12969 * [ES_Count_aaaC] + 0.34282 * [ES_Count_aasN] + 0.12634 * [ES_Count_aasC] - 0.20276 * [ES_Count_dCH2] - 0.18992 * [ES_Count_dNH] + 0.013816 * [IsChiral] + 0.31538 * [Molecular_FractionalPolarSurfaceArea] + 0.0057824 * [Molecular_PolarSurfaceArea] + 0.0036559 * [Molecular_Weight] + 0.31982 * [Num_AromaticRings] + 0.12005 * [Num_H_Acceptors] + 0.062429 * [Num_H_Donors] + 0.35819*[Num_RingAssemblies] + 0.058805 * [Num_RingBonds] + 0.298 * [Num_Rings] + 0.060818 * [Num_RotatableBonds] - 1.4213 * [QED]
PC2	PC2 = -1.1489 - 8.4497e-002 * [ES_Count_aaCH] - 8.5e-002 * [ES_Count_aaN] - 0.23316 * [ES_Count_aaO] + 0.031518 * [ES_Count_aaNH] + 0.17098 * [ES_Count_aaS] - 0.11733 * [ES_Count_aaaC] - 0.40451 * [ES_Count_aasN] - 2.5836e-003 * [ES_Count_aasC] + 0.27566 * [ES_Count_dCH2] + 0.58965 * [ES_Count_dNH] + 0.63983 * [IsChiral] + 4.284 * [Molecular_FractionalPolarSurfaceArea] + 0.012037 * [Molecular_PolarSurfaceArea] + 0.0017616 * [Molecular_Weight] - 0.2058 * [Num_AromaticRings] + 0.18937 * [Num_H_Acceptors] + 0.29776 * [Num_H_Donors] - 0.17029 * [Num_RingAssemblies] - 2.0855e-002 * [Num_RingBonds] - 0.12598 * [Num_Rings] + 0.058959 * [Num_RotatableBonds] - 0.98582 * [QED]
PC3	PC3] = -1.0534 - 2.4072e-002 * [ES_Count_aaCH] + 0.1826 * [ES_Count_aaN] + 0.31781 * [ES_Count_aaO] - 1.5987e-002 * [ES_Count_aaNH] + 1.0455 * [ES_Count_aaS] + 0.15607 *

	[ES_Count_aaaC] + 1.3852 * [ES_Count_aasN] + 0.0092361 * [ES_Count_aasC] - 0.56424 * [ES_Count_dCH2] + 0.28036 * [ES_Count_dNH] - 1.3153 * [IsChiral] + 4.9915 * [Molecular_FractionalPolarSurfaceArea] + 0.0054376 * [Molecular_PolarSurfaceArea] - 2.8807e-003 * [Molecular_Weight] + 0.17648 * [Num_AromaticRings] - 2.4264e-002 * [Num_H_Acceptors] - 7.2115e-002 * [Num_H_Donors] - 1.7229e-002 * [Num_RingAssemblies] - 1.3425e-002 * [Num_RingBonds] - 1.5233e-002 * [Num_Rings] - 0.10781 * [Num_RotatableBonds] + 0.9074 * [QED]

Table S4. Predicted target inhibitory activity of the Bayesian classifier for the 450 molecules with top Libdock scores.

Molecule Name	Predicted Rusult	Bayesian score	Libdock score
4-tert-butyl-N-{2-[(2-{[2-(4-chlorophenoxy)ethyl]amino}-2-oxoethyl)sulfanyl]-1,3-benzothiazol-6-yl}benzamide	A	-8.65927	118.664
N-(2-furylmethyl)-2-naphthalenesulfonamide	A	-7.69051	112.0628
N-(4-ethoxyphenyl)-4-fluorobenzenesulfonamide	A	-7.48884	101.0965
butyl 3-(5-bromo-1,3-dioxo-1,3-dihydro-2H-isoindol-2-yl)benzoate	A	-7.04522	114.179
1-cyclopropyl-3-(4-methoxyphenyl)-2-propen-1-one	A	-6.91184	105.3324
N-[3-(1H-imidazol-1-yl)propyl]-3-methylbenzamide	A	-6.09827	93.8157
N-(2,4-dichlorophenyl)-2-(4-fluorophenoxy)acetamide	A	-6.04216	101.2982
N-[2-(2-benzylphenoxy)ethyl]-3,4,5-trimethoxybenzamide	A	-6.02644	93.447
5-(2-chlorobenzylidene)-2-(4-methyl-1-piperidinyl)-1,3-thiazol-4(5H)-one	A	-6.00667	114.3224
2-butenyl 4-(3-chlorophenyl)-6-methyl-2-oxo-1,2,3,4-tetrahydro-5-pyrimidinecarboxylate	A	-5.98574	101.1396
2-(ethylsulfanyl)-4-(2-methoxyphenyl)-6-oxo-1,4,5,6-tetrahydro-3-pyridinecarbonitrile	A	-5.97073	113.4776
2-{[6-(acetylamino)-1,3-benzothiazol-2-yl]sulfanyl}-N-mesitylacetamide	A	-5.82254	110.0725

N-(2-fluorophenyl)-2-thiophenecarboxamide	A	-5.80953	104.8446
N'-octanoylmethanesulfonohydrazide	A	-5.75607	102.2001
1-(4-benzylpiperazin-1-yl)-3-([1,1'-biphenyl]-4-yloxy)propan-2-ol	A	-5.38767	129.364
2-[(2-methylbenzyl)sulfanyl]-6-(2-thienyl)nicotinonitrile	A	-5.35327	111.6995
2-(4-methoxyphenoxy)-N-(3-methylphenyl)acetamide	A	-5.10047	95.2908
2-(ethylsulfanyl)-6-thien-2-yl-4-(trifluoromethyl)nicotinonitrile	A	-5.09065	106.4048
butyl 6-bromo-2-phenyl-4-quinolinecarboxylate	A	-5.07798	103.4008
N-butyl-1,2-benzisothiazol-3-amine 1,1-dioxide	A	-5.03296	114.7245
N'-(3,4-dichlorobenzylidene)-2-(2-pyridinylsulfanyl)acetohydrazide	A	-4.99747	91.7512
N-(4-bromophenyl)-2-[(4-chlorobenzyl)sulfanyl]acetamide	A	-4.82811	100.9699
1-(3-chlorophenyl)-4-(2-methylbenzoyl)piperazine	A	-4.81925	92.6635
2-(2-chloro-5-iodophenyl)-4-(2,4-dichlorobenzylidene)-1,3-thiazol-5(4H)-one	A	-4.2755	116.5633
2,4-dimethoxybenzaldehyde [4-(4-morpholinyl)-6-(4-toluidino)-1,3,5-triazin-2-yl]hydrazone	A	-4.26071	96.026
5-(2,3-dichlorobenzylidene)-2-thioxo-3-(3-toluidinomethyl)-1,3-thiazolidin-4-one	A	-4.25818	105.8951
3-hexyl-5-methyl-5-[2-(4-toluidino)-1,3-thiazol-4-yl]dihydro-2(3H)-furanone	A	-4.21898	97.3501
2-phenylethyl 4-(3-bromo-5-ethoxy-4-hydroxyphenyl)-1,6-dimethyl-2-oxo-1,2,3,4-tetrahydro-5-pyrimidinecarboxylate	A	-4.21001	115.1746
2-(4-bromophenoxy)-N'-(2,4-dipropoxybenzylidene)acetohydrazide	A	-4.05186	93.1832
2-(1,3-benzothiazol-2-ylsulfanyl)-N'-[(5-methyl-2-furyl)methylene]acetohydrazide	A	-4.03903	91.5326
2-chloro-N-[2-({2-oxo-2-[(1-phenylethyl)amino]ethyl}sulfanyl)-1,3-benzothiazol-6-yl]benzamide	A	-4.00958	118.925
N'-[(5-bromo-2-thienyl)methylene]-2-(3-chlorophenoxy)propanohydrazide	A	-4.0061	100.8421
1-(2-oxo-2-phenylethyl)-3-(2-thiiranylmethyl)-1,3-dihydro-2H-benzimidazol-2-one	A	-3.99293	103.0996

1-(butyrylsulfanyl)-8-methoxy-4,4-dimethyl-4H,5H-[1,2]dithiolo[3,4-c]quinolin-2-ium	A	-3.93547	87.5752
N-(2,5-dimethoxyphenyl)-4-fluorobenzenesulfonamide	A	-3.91137	113.0395
2-methoxybenzaldehyde [3-(trifluoromethyl)[1,2,4]triazolo[4,3-b]pyridazin-6-yl]hydrazone	A	-3.90878	111.951
N-[1-({2-[(6-methoxy-2-naphthyl)methylene]hydrazino}carbonyl)-2-(4-propoxyphenyl)vinyl]benzamide	A	-3.89909	106.687
N-(5-methyl-1,3,4-thiadiazol-2-yl)-3-phenylpropanamide	A	-3.89428	95.541
4-methyl-5-[(2-methylphenoxy)methyl]-4H-1,2,4-triazol-3-yl hydrosulfide	A	-3.89331	115.23
2-[(4-chlorobenzyl)sulfanyl]-N-(4-methylphenyl)acetamide	A	-3.83439	91.5119
5-{[3-(ethoxycarbonyl)-4,5-dimethyl-2-thienyl]amino}-5-oxopentanoic acid	A	-3.83162	107.762
N-(4-ethoxyphenyl)-2-thiophenecarboxamide	A	-3.82818	91.7835
N'-cycloheptylidene-2-[(4,6-dimethyl-2-pyrimidinyl)sulfanyl]acetohydrazide	A	-3.82736	86.9994
3-cyclopentyl-N-(3-pyridinyl)propanamide	A	-3.8251	97.9751
2-[(2,6-dichlorobenzylidene)amino]-5,6-dihydro-4H-cyclopenta[b]thiophene-3-carbonitrile	A	-3.82425	108.6155
5-chloro-2-(2-methylphenyl)-1,3-benzoxazole	A	-3.82111	111.203
4-(dodecylamino)-1-oxaspiro[4.5]dec-3-en-2-one	A	-3.81931	100.164
2-(ethylsulfanyl)ethyl 4-(4-hydroxy-3-methoxyphenyl)-2,7,7-trimethyl-5-oxo-1,4,5,6,7,8-hexahydro-3-quinolinecarboxylate	A	-3.8182	110.0165
1-(2-chlorobenzyl)-1H-benzimidazole-2-thiol	A	-3.81301	107.6238
2-[(2-fluorobenzyl)sulfanyl]-4,6-dithien-2-ylnicotinonitrile	A	-3.81277	110.3453
2-(2,4-dichlorophenoxy)-N'-(2,4-dipropoxybenzylidene)acetohydrazide	A	-3.80822	112.8895
N-(4-chlorophenyl)-3-(1-pyrrolidinyl)propanamide	A	-3.80365	114.7359
2-[(5-methyl-1,3,4-thiadiazol-2-yl)sulfanyl]-N-(1,3-thiazol-2-yl)acetamide	A	-3.80028	103.089
5-(5-bromo-2-isopropoxybenzylidene)-3-phenyl-2-(phenylimino)-1,3-thiazolidin-4-one	A	-3.79129	110.3162

N'-benzylidene-2-[4-(benzyloxy)phenoxy]propanohydrazide	A	-3.79073	96.7657
5-{2-[(2-chlorobenzyl)oxy]-3-methoxybenzylidene}-3-phenyl-2-(phenylimino)-1,3-thiazolidin-4-one	A	-3.78874	105.5763
3,4,5-trimethoxy-N-(tetrahydro-2-furanylmethyl)benzamide	A	-3.78117	105.8894
1-{7-acetyl-9-[4-(octyloxy)benzylidene]-9H-fluoren-2-yl}ethanone	A	-3.72027	127.735
5-[2-(2-chloroanilino)-1,3-thiazol-4-yl]-3-hexyl-5-methyldihydro-2(3H)-furanone	A	-3.66547	108.8804
ethyl 2-{[(cyanosulfanyl)acetyl]amino}-4,5,6,7-tetrahydro-1-benzothiophene-3-carboxylate	A	-3.66445	102.091
3-[2-[(4-chlorobenzyl)oxy]-4-(diethylamino)phenyl]-2-(5-methyl-1H-benzimidazol-2-yl)acrylonitrile	A	-3.66278	115.2335
N'-{[5-(1H-benzimidazol-2-ylsulfanyl)-2-furyl]methylene}-4-(decyloxy)benzohydrazide	A	-3.57015	106.9132
4-{4-[(2-chlorobenzyl)oxy]-3-ethoxybenzylidene}-2-phenyl-1,3-oxazol-5(4H)-one	A	-3.5615	120.736
N-[2-(2-hydroxyethoxy)ethyl][1,1'-biphenyl]-4-carboxamide	A	-3.56138	105.1815
2-[(2-fluorobenzoyl)amino]-N-(3-methylphenyl)-4,5,6,7-tetrahydro-1-benzothiophene-3-carboxamide	A	-3.56067	107.455
2-[(1H-benzimidazol-2-ylmethyl)sulfanyl]-6-ethoxy-1,3-benzothiazole	A	-3.51163	106.1983
2-chlorobenzyl 4-methyl-4H-1,2,4-triazol-3-yl sulfide	A	-3.51079	102.5039
ethyl 4-(2-(4-isobutoxyphenyl)-4-oxo-1,4-dihydro-3(2H)-quinazolinyl)benzoate	A	-3.50682	94.2418
2-[(2-fluorobenzyl)sulfanyl]-3-methyl-4(3H)-quinazolinone	A	-3.5045	107.7533
2-[(6-ethoxy-1,3-benzothiazol-2-yl)sulfanyl]-N-(4-fluorophenyl)acetamide	A	-3.50433	115.3984
3-allyl-5-{4-[(2-fluorobenzyl)oxy]-3-methoxybenzylidene}-2-thioxo-1,3-thiazolidin-4-one	A	-3.49962	112.0049
6-(4-(3-fluoro-4-methoxyphenyl)-2-{[3-(trifluoromethyl)phenyl]imino}-1,3-thiazol-3(2H)-yl)-1-hexanol	A	-3.49892	117.047
3-(1,3-benzothiazol-2-yl)-N-(3-pyridinylmethyl)aniline	A	-3.46167	111.353
N-(3-cyano-4,5,6,7-tetrahydro-1-benzothien-2-yl)-2-phenylacetamide	A	-3.38473	97.968
3-(4-sec-butylphenyl)-2-(4-propoxyphenyl)-2,3-dihydro-4(1H)-quinazolinone	A	-3.38027	94.4639

2-(methylsulfanyl)ethyl 6-methyl-2-oxo-4-(4-propoxyphenyl)-1,2,3,4-tetrahydro-5-pyrimidinecarboxylate	A	-3.21281	106.2162
4-chlorobenzyl 1-(2,4-dichlorophenyl)-2-(1H-imidazol-1-yl)ethyl ether	A	-3.21217	116.827
8-quinolinyl 2-thiophenecarboxylate	A	-3.21069	114.2214
1-(4-phenyl-1-buten-3-ynyl)naphthalene	A	-3.20935	95.0491
2-methyl-1-(4-phenyl-1-buten-3-ynyl)naphthalene	A	-3.15526	97.9548
4-[(1-naphthylmethylene)amino]phenyl 4-methoxybenzenesulfonate	A	-3.1541	92.9497
2-(4-phenyl-1-buten-3-ynyl)benzo[c]phenanthrene	A	-3.14875	105.3658
3-(4-phenyl-1-buten-3-ynyl)phenanthrene	A	-3.04366	104.6039
4-{(2,5-dioxo-1-phenyl-3-pyrrolidinyl)[2-(4-methoxyphenyl)ethyl]amino}-4-oxo-2-butenoic acid	A	-3.04157	120.938
2-oxo-2-phenylethyl (3,4-dimethylphenoxy)acetate	A	-3.0393	105.1525
2-[2-(3,5-dimethylphenyl)vinyl]naphthalene	A	-3.02508	107.5743
2-methyl-N-(8-quinolinyl)benzenesulfonamide	A	-3.02278	103.114
2-(4-chlorobenzoyl)-N-(2-pyridinyl)benzamide	A	-3.02112	115.8744
methyl 2-{2-nitroanilino}benzoate	A	-3.02023	101.9154
ethyl 5-nitro-3-{[(2-phenylethyl)sulfanyl]methyl}-1-benzothiophene-2-carboxylate	A	-3.01755	109.8901
N-(6-methylpyridin-2-yl)nicotinamide	A	-3.01532	90.6558
2,2-dimethylhexahydrofuro[2',3':4,5]furo[2,3-d][1,3]dioxol-6-ol	A	-3.0134	89.8406
ethyl2-({4-[(dipropylamino)sulfonyl]benzoyl}amino)-5-ethyl-3-thiophenecarboxylate	A	-3.01329	123.222
1,3-dimethyl-5-(4-phenyl-1-buten-3-ynyl)benzene	A	-3.01131	106.7781
N-(9H-fluoren-2-yl)-4-methoxybenzamide	A	-2.99658	93.6782
3-allyl-2-({2-[1-(2-methoxyethyl)-2,5-dimethyl-1H-pyrrol-3-yl]-2-oxoethyl}sulfanyl)-3,5,6,7-tetrahydro-4H-cyclopenta[4,	A	-2.9958	133.492

5]thieno[2,3-d]pyrimidin-4-one			
2,2,2-trifluoro-N-(1,3-thiazol-2-yl)acetamide	A	-2.99567	89.24<br>71
4-chlorophenyl 3-{4-nitrophenyl}acrylate	A	-2.9909	90.93<br>43
4-bromo-2-[(3,4-dimethylanilino)methyl]phenol	A	-2.98792	91.19<br>38
2-methoxyphenyl 2-thiophenecarboxylate	A	-2.98545	100.9<br>298
2-(1,3-dithiolan-2-ylidene)-5-(3-hydroxyphenyl)-3-oxo-4-pe<br>ntenoic acid	A	-2.98186	93.05<br>87
N-methyl-4-phenyl-1,3-thiazol-2-amine	A	-2.98163	89.23<br>94
2-methoxy-4-(2-phenylcarbohydrazonoyl)phenyl<br>3,4-dimethoxybenzoate	A	-2.98074	107.3<br>59
ethyl 3-(4-chlorophenyl)-2-(phenylsulfonyl)acrylate	A	-2.9803	92.10<br>83
1-bromo-3-[(phenylsulfonyl)methyl]spiro(pentacyclo[3.3.1.0<br>~2,4~.0~3,7~.0~6,8~]nonane-9,2'-[1,3]-dioxolane)	A	-2.97743	103.8<br>132
2-[6-(2-thienyl)-2-naphthyl]thiophene	A	-2.97461	100.4<br>042
[3-(1,3-dioxo-1,3-dihydro-2H-isoindol-2-yl)-4-methoxy-4-ox<br>obutyl](dimethyl)sulfonium	A	-2.97408	104.0<br>56
4-bromo-N-[2-(2-cyclooctylidenehydrazino)-2-oxoethyl]-N-(<br>4-ethoxyphenyl)benzenesulfonamide	A	-2.97368	121.4<br>55
3-oxa-4-azatricyclo[5.2.1.0~2,6~]dec-4-en-5-yl(phenyl)metha<br>none	A	-2.97189	97.35<br>37
3-(4-toluidinocarbonyl)-7-oxabicyclo[2.2.1]hept-5-ene-2-carb<br>oxylic acid	A	-2.89157	109.8<br>405
diethyl 4-oxo-4H-pyran-2,6-dicarboxylate	A	-2.88905	99.82<br>07
2-(4-chlorobenzoyl)-N-(4-methoxyphenyl)benzamide	A	-2.888	110.5<br>849
N-methyl-N-phenyl-2-thiophenecarboxamide	A	-2.80493	93.74<br>64
N-(1,3-thiazol-2-yl)acetamide	A	-2.80394	86.12<br>24
2-pyridinyl 4-bromobenzenesulfonate	A	-2.80268	91.81<br>16
5-[4-(benzyloxy)-3-methoxyphenyl]-4-(2,3-dihydro-1,4-benz<br>odioxin-6-ylcarbonyl)-3-hydroxy-1-(3-pyridinylmethyl)-1,5-<br>dihydro-2H-pyrrol-2-one	A	-2.80268	156.5<br>2
(4-phenyl-3-buten-1-ynyl)benzene	A	-2.80211	86.56<br>03

N-(2-naphthyl)nicotinamide	A	-2.79993	89.97 45
4-propyl-1-cubanecarboxylic acid	A	-2.79837	87.81 02
2-{[(2-chloro-3-pyridinyl)amino]carbonyl}benzoic acid	A	-2.79677	104.6 12
benzaldehyde (2,6-dioxo-1,2,3,6-tetrahydro-4-pyrimidinyl)hydrazone	A	-2.77325	99.33 64
ethyl 2-(benzoylamino)benzoate	A	-2.77185	86.79 48
[({[(3-chloro-1-benzothien-2-yl)carbonyl]amino}carbothioyl)amino]acetic acid	A	-2.67481	109.6 558
N-[2-(cyclohexylsulfanyl)-5-nitrobenzylidene](phenyl)methanamine	A	-2.66921	103.7 35
2-chloro-2-(2-iodophenyl)-N,N-dimethylethanamine	A	-2.65316	102.6 508
4-phenacylpyridine	A	-2.6393	99.74 75
dimethyl 2-(4-methoxybenzyl)-2-[2-(4-methoxybenzyl)-2-propenyl]malonate	A	-2.63744	106.6 707
3-[(4-methoxyanilino)carbonyl]-7-oxabicyclo[2.2.1]hept-5-ene-2-carboxylic acid	A	-2.58719	108.8 018
4,5-dibromotricyclo[5.2.1.0~2,6~]deca-4,8-dien-3-one	A	-2.58487	107.5 657
4-(2,4-difluoroanilino)-4-oxobutanoic acid	A	-2.5832	98.00 18
4-{[4-(dimethylamino)-2-methylphenyl]diazenyl}benzonitrile	A	-2.58092	110.5 901
4-oxo-4-(2-thienyl)butanoic acid	A	-2.57895	100.7 065
4-{[2-ethoxy-1-({4-nitrophenyl}diazenyl)-2-oxoethyl]sulfanyl}butanoic acid	A	-2.57785	94.69 44
(3-chloro-1-benzothien-2-yl)methanol	A	-2.56028	106.0 912
methyl 4-(acetylamino)-3-thiophenecarboxylate	A	-2.55971	96.52 93
3-methyl-2-butenylphenylcarbamate	A	-2.55936	92.23 45
3-(3-bromophenyl)-N-cyclopropylacrylamide	A	-2.55843	103.6 477
(2-phenylethyl)benzene	A	-2.55515	94.64 38
1-(1-benzothien-3-yl)-2-(diethylamino)ethanone	A	-2.55476	107.2

			867
[bis(benzylthio)methyl]benzene	A	-2.53902	92.7597
1-(benzyloxy)-4-methoxybenzene	A	-2.53581	89.242
ethyl 3-[(diethylamino)methyl]-1-benzothiophene-5-carboxylate	A	-2.51583	102.847
N-(2-iodophenyl)-2-adamantanecarboxamide	A	-2.51119	98.744
(5-{[(4-chlorobenzoyl)oxy]methyl}-1,3-dioxan-5-yl)methyl 4-chlorobenzoate	A	-2.51049	117.047
4-methyl-N-(1,3-thiazol-2-yl)benzenesulfonamide	A	-2.48803	106.596
2-nitro-N-phenyl-1-benzothiophen-3-amine	A	-2.48468	95.685
1-benzoylamino-2,4-dimethyl-benzene	A	-2.422	84.344
4-ethoxybenzaldehyde O-(4-chlorobenzoyl)oxime	A	-2.41951	100.472
N-(4-tetradecylphenyl)acetamide	A	-2.39339	100.3229
O,O-diphenyl thiocarbonate	A	-2.39332	101.4888
2-chloro-3-(phenylsulfonyl)propanenitrile	A	-2.39327	114.4836
2-(dimethylamino)-1-(2-iodophenyl)ethanol	A	-2.39251	91.919
2-phenoxy-4-pyrimidinamine	A	-2.38993	109.6356
2-{2-[(hydroxyimino)methyl]anilino}acetamide	A	-2.3895	112.8286
9-[4-methoxy-3-(1-pyrrolidinylsulfonyl)phenyl]-3,4,5,6,7,9-hexahydro-1H-xanthene-1,8(2H)-dione	A	-2.38826	90.9059
(5-iodo-1-benzothien-2-yl)methanamine	A	-2.37105	90.3814
2-methoxy-4-(2-phenylcarbohydrazonoyl)phenyl 3-(4-methoxyphenyl)acrylate	A	-2.37084	113.793
[4-(1-propenylidene)cyclohexyl]benzene	A	-2.36885	110.672
2,2'-bis[2-(4-methoxyphenyl)vinyl]-1,1'-biphenyl	A	-2.36807	110.0317
2-[2-(4-methoxyphenyl)vinyl]-1,1'-biphenyl	A	-2.36724	96.1056
cholest-4-ene-3,6-dione 3-oxime	A	-2.36681	111.2

			514
1-methyl-2-oxo-1,2-dihydro-4-quinolinyl 2-methylbenzoate	A	-2.36657	103.0701
5-tert-butyl-2,3-dihydro[1,3]thiazolo[2,3-b][1,3]thiazol-4-ium	A	-2.36254	91.4464
diethyl 2-(benzoylamino)malonate	A	-2.36253	102.2789
2-hydroxy-4,6-diphenylpyrimidine	A	-2.35889	98.8779
methyl 2-(cyanomethoxy)benzoate	A	-2.35298	88.328
cyclohexanone O-{4-nitrophenyl}oxime	A	-2.34301	110.9882
methyl 3-bromo-2-pyridinecarboxylate	A	-2.34022	86.5645
6,7,8,9,10,11,12,13-octahydro-5H-cyclodeca[b]indole	A	-2.33922	100.1744
2-fluoro-N,N-dimethyl-2-phenylacetamide	A	-2.33752	98.4332
1-(5-bromo-1-benzothien-2-yl)-2-(diethylamino)ethanol	A	-2.33731	109.3704
2-[(3-bromobenzylidene)amino]-1H-benzo[de]isoquinoline-1,3(2H)-dione	N	-2.33577	98.5371
5,7-dibromo-8-quinolinyl 3-chlorobenzoate	N	-2.33563	98.0176
N'-(1-naphthylmethylene)-3-phenylpropanohydrazide	N	-2.33297	86.4004
N'-(2,4-dihydroxybenzylidene)-2-phenoxyacetohydrazide	N	-2.30512	101.2572
3-[(3-oxo[1,3]thiazolo[3,2-a]benzimidazol-2(3H)-ylidene)methyl]phenyl acetate	N	-2.30391	85.851
3-{2-[(2-methylphenoxy)acetyl]carbohydrazonoyl}phenyl 3-fluorobenzoate	N	-2.29783	92.2749
N-(8-quinolinyl)undecanamide	N	-2.29631	102.9733
4-phenyl-N-(1,3-thiazol-2-yl)butanamide	N	-2.29502	101.8464
N'-[(2-bromo-1-benzothien-3-yl)methylene]-2-hydroxybenzohydrazide	N	-2.29348	100.4361
2-oxo-2-phenylethyl 3-chlorobenzoate	N	-2.2901	99.8834
7-chloro-2-{3-nitrophenyl}-1-(4-methylbenzoyl)-1,2-dihydropyrrolo[1,2-a]quinoline-3,3(3aH)-dicarbonitrile	N	-2.28867	99.7154
2-[(1-bromo-2-naphthyl)oxy]-N'-(2,3-dimethoxybenzylidene	N	-2.28792	97.00

)butanohydrazide			19
3-[(2-chlorophenyl)imino]-2-nitropropanal	N	-2.14359	98.40<br>12
2,4-dibromo-6-({[2-(2-furyl)imidazo[1,2-a]pyridin-3-yl]imin<br>o}methyl)phenol	N	-2.14077	111.4<br>47
N-(3-methylphenyl)-1-naphthamide	N	-2.13983	107.4<br>55
N-(2-methoxyphenyl)tricyclo[3.2.1.0~2,4~]octane-3-carboxa<br>mide	N	-2.13461	85.68<br>95
4-chloro-2-({[2-(4-chlorophenoxy)-5-(trifluoromethyl)phenyl<br>]imino}methyl)phenol	N	-2.11515	91.75<br>25
ethyl<br>2-[(2,3-dihydro-1,4-benzodioxin-6-ylcarbonyl)amino]-5-ethy<br>l-3-thiophenecarboxylate	N	-2.11278	104.1<br>08
2-hydroxy-3,5-diiodobenzaldehyde<br>(4-(1-azepanyl)-6-{4-nitroanilino}-1,3,5-triazin-2-yl)hydrazo<br>ne	N	-2.10945	103.7<br>39
N-(3-bromophenyl)-4-phenylbutanamide	N	-2.10614	78.33<br>65
N-(1-adamantyl)-3-{[(4-bromo-2-methylphenoxy)acetyl]hyd<br>razono}butanamide	N	-2.10548	92.70<br>15
allyl 2-amino-4-methyl-1,3-thiazole-5-carboxylate	N	-2.09464	98.15<br>36
ethyl {4-nitro-1H-pyrazol-1-yl}acetate	N	-2.09431	97.64<br>15
N'~1~,N'~2~-bis(3-bromobenzylidene)-1,2-cyclopropanedica<br>rbohydrazide	N	-2.09429	104.6<br>924
N-(5-chloro-2-pyridinyl)-2-methylbenzamide	N	-2.09424	93.02<br>38
N'-acetyl-3-iodobenzohydrazide	N	-2.09417	87.38<br>76
1-[(2,3-dichlorophenyl)sulfonyl]piperidine	N	-2.09398	104.7<br>269
4-bromo-N'-(3-iodo-4-methoxybenzylidene)benzohydrazide	N	-2.08819	101.2<br>538
N-[3-(dimethylamino)propyl]-3-hydroxy-2-naphthamide	N	-2.08672	112.1<br>791
O-{3-[(3-bromoanilino)carbonyl]phenyl}<br>1-piperidinecarbothioate	N	-2.0859	108.9<br>554
N-(5-ethyl-1,3,4-thiadiazol-2-yl)hexanamide	N	-2.0809	104.0<br>655
2-[(3-bromobenzyl)oxy]-N'-(2,4-dipropoxybenzylidene)ben<br>zohydrazide	N	-2.06272	89.15<br>22
ethyl	N	-2.06187	93.83

2-({4-[(dipropylamino)sulfonyl]benzoyl}amino)-5-ethyl-3-th iophenecarboxylate			39
4-(dimethylamino)-2-methyl-1-phenyl-2-butanol	N	-2.03914	94.30 85
2-{[2-(3-phenylpropanoyl)hydrazino]carbonyl}benzoic acid	N	-2.01955	103.2 22
N-{2-oxo-2-[2-(1H-pyrrol-2-ylmethylene)hydrazino]ethyl}-2 -thiophenecarboxamide	N	-2.01903	86.88 58
N-(5-chloro-2-pyridinyl)-4-ethylbenzenesulfonamide	N	-2.0187	107.0 417
N'-({5-nitro-2-thienyl}methylene)-2-(3-methylphenoxy)aceto hydrazide	N	-2.0037	108.7 411
ethyl 5-octyl-1,3,4-thiadiazol-2-ylcarbamate	N	-2.00294	88.01 94
5-(3-methoxyphenyl)-1,3,4-oxadiazole-2-thiol	N	-2.00191	87.92 3
4-{(2,5-dioxo-1-phenyl-3-pyrrolidinyl)[2-(4-methoxyphenyl) ethyl]amino}-4-oxo-2-butenoic acid	N	-1.98462	109.6 855
2-(4-butylphenoxy)-N'-[(5-methyl-2-furyl)methylene]acetoh ydrazide	N	-1.98348	114.2 543
2-(1H-1,2,3-benzotriazol-1-yl)-N'-(2-fluorobenzylidene)acet ohydrazide	N	-1.98171	122.0 93
2-(1-piperidinyl)-N-(1,3-thiazol-2-yl)acetamide	N	-1.97927	98.50 06
7-(allyloxy)-2,3-dihydrocyclopenta[c]chromen-4(1H)-one	N	-1.97886	94.97 58
4-fluoro-N-(1,3-thiazol-2-yl)benzenesulfonamide	N	-1.97863	113.9 38
N-[4-(acetylamino)phenyl]-2-(benzylsulfanyl)benzamide	N	-1.97573	98.78 1
3-bromobenzaldehyde [4-(2-oxo-2H-chromen-3-yl)-1,3-thiazol-2-yl]hydrazone	N	-1.966	94.88 27
N'-[(5-bromo-2-thienyl)methylene]-2-(3-methylphenoxy)ace tohydrazide	N	-1.96564	102.2 237
tert-butyl 2-[3-(cyclopentylamino)-1-methyl-3-oxopropylidene]hydraz inecarboxylate	N	-1.96512	93.07 68
N-(2-methoxyphenyl)pentanamide	N	-1.96496	94.66 17
4-tert-butyl-N-(2-pyridinylmethyl)benzamide	N	-1.96442	95.39 39
N-(4-cyanophenyl)-2,2,2-trifluoroacetamide	N	-1.96417	85.96 16
2-bromo-N'-[3-oxo-3-(2-thienyl)-1-(trifluoromethyl)propylid	N	-1.93312	95.96

ene]benzohydrazide			9
N'-(2-chlorobenzylidene)-3-(2-methylphenoxy)propanohydrazide	N	-1.9329	96.2113
N-(2-ethoxyphenyl)-5-methyl-2-furamide	N	-1.93144	108.6912
1-phenyl-4-(1-pyrrolidinyl)-2-butyn-1-ol	N	-1.92926	99.2289
3-phenyl-N-(3-pyridinyl)propanamide	N	-1.92821	90.0508
ethyl 4-(1-chloro-1-decenyl)-3,5-dimethyl-1H-pyrrole-2-carboxylate	N	-1.92662	85.2625
8-[(2-phenoxyethyl)sulfanyl]-9H-purin-6-amine	N	-1.7536	92.6671
7-[2-(4-methoxyphenyl)-2-oxoethoxy]-4-methyl-2H-chromen-2-one	N	-1.7521	102.2741
5-(2-chlorophenyl)-2-(3,4-dichlorobenzyl)-2H-tetraazole	N	-1.75064	86.7628
4-(4-cyanoanilino)-4-oxobutanoic acid	N	-1.74789	94.4591
5-[(2,3-dimethylphenoxy)methyl]-1,3,4-thiadiazol-2-amine	N	-1.74691	86.893
N'-[1-(3-aminophenyl)ethylidene]-2-phenoxyacetohydrazide	N	-1.74581	88.1319
N-(2,4-dimethylphenyl)-2-(4-methylphenoxy)acetamide	N	-1.7307	88.2733
2-(1,3-benzothiazol-2-ylsulfanyl)-N'-(2-thienylmethylene)acetohydrazide	N	-1.72904	88.8447
N-(1,3-benzothiazol-2-yl)-4-phenylbutanamide	N	-1.72865	89.5961
2-chloro-N-{3-[(2-iodobenzoyl)amino]phenyl}benzamide	N	-1.7271	89.8581
4-{3-[(5-bromo-2-isopropoxybenzylidene)amino]imidazo[1,2-a]pyridin-2-yl}-2-methoxyphenol	N	-1.72691	90.1108
N-(4-ethoxyphenyl)-2-(4-fluorophenoxy)acetamide	N	-1.72527	90.9126
2-chloro-N-{3-[(4-pyridinylmethyl)amino]phenyl}benzamide	N	-1.72425	93.3098
3-[(3-bromoanilino)methyl]-5-(4-tert-butylphenyl)-1,3,4-oxadiazole-2(3H)-thione	N	-1.7237	93.5204
1,4-bis(2,3-difluorobenzoyl)piperazine	N	-1.72336	101.1892
N-(1,3-benzothiazol-2-yl)-2-(4-fluorophenoxy)acetamide	N	-1.72285	101.1919

N'-(1,3-diphenyl-2-propenylidene)-2-phenoxyacetohydrazide	N	-1.72038	93.7087
5-(benzylamino)-4-chloro-2-phenyl-3(2H)-pyridazinone	N	-1.71914	94.5685
N'-[5-methoxy-2-(pentyloxy)benzylidene]-2-(phenylsulfanyl)acetohydrazide	N	-1.71849	100.6958
5-(3-bromo-4-methoxybenzylidene)-2-[(3-methylphenyl)imino]-1,3-thiazolidin-4-one	N	-1.70009	100.9889
2-bromo-N-[3-(4-morpholinyl)propyl]benzamide	N	-1.69111	107.704
N'-benzylidene-2-(2-methoxyanilino)acetohydrazide	N	-1.6897	97.9028
3-[(2,4-dimethylphenyl)imino]-1-[(4-methyl-1-piperidinyl)methyl]-1,3-dihydro-2H-indol-2-one	N	-1.68947	121.33
4-(2-butoxybenzylidene)-2-(4-methylphenyl)-1,3-oxazol-5(4H)-one	N	-1.68891	103.6824
2-cyano-2-{[3-(trifluoromethyl)phenyl]hydrazono}ethanethioamide	N	-1.68016	95.6634
2-[[(5-bromo-1-benzothien-3-yl)methyl](ethyl)amino]ethanol	N	-1.67958	92.6907
7-chloro-2-(2-hydroxyethyl)-2-methyl-2,3-dihydro-1H-[1]benzothieno[2,3-c]pyrrol-2-ium	N	-1.67886	101.5023
7-methoxy-3-(4-methoxyphenyl)-2,3-dihydro-4H-chromen-4-one	N	-1.67522	98.7093
5-(benzyloxy)-6-phenyl-3(2H)-pyridazinone	N	-1.67494	102.4841
5-phenyl-2-(sulfanylmethyl)-4,6-pyrimidinediol	N	-1.66915	98.3613
5-[2,5-dibutoxy-4-(5'-phenyl-5,2'-bithien-2-yl)phenyl]-5'-phenyl-2,2'-bithiophene	N	-1.66461	86.7601
(4-pentylbicyclo[2.2.2]oct-1-yl)methyl 4-methylbenzenesulfonate	N	-1.6629	92.1612
5-(hydroxyimino)-5-(3-pyridinyl)pentanoic acid	N	-1.6618	104.7482
2-(cyclohexylamino)-N-(3,5-dichlorophenyl)-2-thioxoacetamide	N	-1.6605	87.8955
ethyl chloro[(4-chlorophenyl)hydrazono]acetate	N	-1.65405	85.9614
2-(4-methoxybenzoyl)-1,2-dihydro-1-isoquinolinecarbonitrile	N	-1.65257	101.9654
methyl 4-morpholinylcarbothioylcarbamate	N	-1.65155	94.6291
4,5-dibromo-3-methyl-1-phenyl-1H-pyrazole	N	-1.64979	98.5219

2-(benzylamino)-N-(4-methylphenyl)-2-thioxoacetamide	N	-1.63943	99.816
1-(4-bromophenyl)-1,3-octanedione	N	-1.63876	90.7937
N-(2-cyanophenyl)-2-[(4-methylphenyl)sulfanyl]acetamide	N	-1.6387	91.3575
4-(benzyloxy)-2-(dimethylamino)-7(8H)-pteridinone	N	-1.63562	109.6266
2-(4-chlorophenoxy)-N-(2-{[(4-chlorophenoxy)acetyl]amino}phenyl)acetamide	N	-1.63363	110.771
4-tert-butyl-3-methyl-1,3-thiazole-2(3H)-thione	N	-1.62712	108.6486
2-(4-pentynyl)-5-phenylpyrimidine	N	-1.6266	114.5006
[4-(isopentyloxy)phenyl]acetic acid	N	-1.6256	114.3214
N-benzoyl-N'-methylthiourea	N	-1.62488	100.4305
5-butyl-2-(2-thienyl)-1,3-dioxane	N	-1.61506	86.1117
2-oxo-1-phenyl-1,2,5,6,7,8-hexahydro-4-quinolinyl 4-methoxybenzoate	N	-1.61456	92.4812
2,2-dimethyl-N-(4-morpholinylcarbothioyl)propanamide	N	-1.61028	93.3863
N'-(4-methoxybenzoyl)-N-methyl-N-phenylthiourea	N	-1.60993	107.925
dibenzyl cyanodithioimidocarbonate	N	-1.60441	98.4932
2-[2,5-dibutoxy-4-(5-iodo-2-thienyl)phenyl]-5-iodothiophene	N	-1.60427	104.119
4'-[(2-pentylcyclopropyl)carbonyl][1,1'-biphenyl]-4-carbonitrile	N	-1.60422	104.9153
N-(1,3-thiazol-2-yl)-2-thiophenecarboxamide	N	-1.5935	111.924
N-{4-chloro-3-nitrophenyl}-3-methylbenzamide	N	-1.59319	104.5117
2-(benzoylamino)-N-(4-methyl-2-pyridinyl)benzamide	N	-1.59221	87.0164
N'-benzoyl-N,N-dimethylthiourea	N	-1.58399	97.4799
4-methyl-N-phenyl-1,3-thiazol-2-amine	N	-1.58316	88.1852
1-ethynylcyclohexyl phenylcarbamate	N	-1.58158	92.9258

4-methoxy-N-phenylbenzenecarbothioamide	N	-1.57813	94.0658
4-(4-methylphenyl)-2,4-dihydro-3H-1,2,4-triazol-3-one	N	-1.57624	91.7596
N,N-dimethyl-N'-(2-thienylcarbonyl)thiourea	N	-1.5757	94.2399
4-methoxy-N-{[2-(phenoxyacetyl)hydrazino]carbothioyl}benzamide	N	-1.56691	118.4814
N-(2-chloro-3-pyridinyl)isonicotinamide	N	-1.56663	112.7779
3-(5-{2-nitrophenyl}-2-furyl)-1-phenyl-2-propen-1-one	N	-1.56663	104.3086
ethyl 1,1,2,2-tetramethyl-3-oxo-1,8b-dihydro-2H-cyclobuta[c]chromene-2a(3H)-carboxylate	N	-1.56407	98.0819
ethyl 4-hydroxy-1-(4-methoxyphenyl)-2-methyl-6-oxo-1,6-dihydro-3-pyridinecarboxylate	N	-1.55549	97.9284
N~6~-[(benzyloxy)carbonyl]lysine	N	-1.55288	113.8226
3-methyl-6-phenyl-7H-imidazo[2,1-b][1,3]thiazol-4-ium	N	-1.55068	115.4153
2-[[(5-chloro-1-benzothien-3-yl)methyl](ethyl)amino]ethanol	N	-1.54272	103.921
2-chloro-N-[(5-iodo-1-benzothien-2-yl)methyl]-N-methylethanamine	N	-1.54229	105.0578
N-cyclohexyl-2-phenylacetamide	N	-1.5412	104.7445
4-tert-butyl-1,3-thiazole-2(3H)-thione	N	-1.53859	100.973
3-[(7-chloro-4-quinolinyl)amino]-1-propanol	N	-1.53853	99.383
2-(4-methoxy-2-methylphenyl)-1-(4-methoxyphenyl)ethanone	N	-1.53528	115.18
({[(benzylthio)methyl]thio}methyl)benzene	N	-1.51994	128.064
1-methyl-4-{[(4-methylbenzyl)sulfanyl]methyl}benzene	N	-1.51937	119.3581
N-(5-chloro-2-methoxyphenyl)benzamide	N	-1.51077	91.4331
(1,3-dioxo-1,3-dihydro-2H-isoindol-2-yl)methyl 2-(4-methoxybenzylidene)hydrazinecarbodithioate	N	-1.50962	98.554
3-(4-chlorophenyl)-3-[(4-chlorophenyl)sulfanyl]-1-cyclopropyl-1-propanone	N	-1.50935	105.1243

N-bicyclo[2.2.1]hept-2-yl-4-methylbenzenesulfonamide	N	-1.50123	105.1305
N'-[3-(trifluoromethyl)benzylidene]-2-thiophenecarbohydrazide	N	-1.50003	112.8239
3-(phenylsulfanyl)-2-cyclohexen-1-one	N	-1.49988	89.708
1,3-dibutyl-4-hydroxy-2(1H)-quinolinone	N	-1.49979	92.5294
2-(benzylamino)-N-(2,3-dimethylphenyl)-2-thioxoacetamide	N	-1.49144	122.407
methyl 5-bromo-2-furoate	N	-1.49143	89.8716
N-benzyl-1-octanamine	N	-1.49078	96.027
diethyl 6-methyl-4-oxo-4H-quinolizine-1,3-dicarboxylate	N	-1.48909	99.8164
2-bromo-6-(phenylsulfanyl)pyridine	N	-1.48894	94.6414
N-(4-morpholinylcarbothioyl)benzamide	N	-1.48033	124.005
N-(1,3-benzothiazol-2-yl)-3-methoxybenzamide	N	-1.29554	104.4653
N-(1-azepanylcarbothioyl)benzamide	N	-1.29536	87.0903
N-(4-methoxyphenyl)-2-[(1-methyl-1H-imidazol-2-yl)sulfanyl]acetamide	N	-1.2947	106.4316
butyl 2-phenylvinyl sulfone	N	-1.29445	97.6455
N-(5-methyl-1,3,4-thiadiazol-2-yl)pentanamide	N	-1.29427	93.9293
6-phenylimidazo[2,1-b][1,3]thiazole	N	-1.29213	101.3064
1-(4-bromophenyl)-1-tetradecanone	N	-1.2916	98.3811
2-(2-imidazolidinylidene)-1-phenylethanone	N	-1.29114	96.1328
5-(benzylideneamino)-6-[(2-hydroxyethyl)amino]-2,4(1H,3H)-pyrimidinedione	N	-1.28974	90.9541
ethyl 4-hexyl-2-methyl-4,5-dihydro-1,3-oxazole-5-carboxylate	N	-1.27321	94.5344
1-benzoylpiperidine	N	-1.27309	96.8048
ethyl {[2-(4-methoxyphenyl)-4-oxo-4H-chromen-3-yl]oxy}acetate	N	-1.27166	90.3048

6-oxo-1,2-diphenyl-1,6-dihydro-4-pyrimidinyl benzoate	N	-1.27118	97.827
N-(4-tert-butyl-1,3-thiazol-2-yl)-2,2,2-trifluoroacetamide	N	-1.26919	98.3486
4'-{[(4,5-dihydroxypentyl)oxy]methyl}[1,1'-biphenyl]-4-carbonitrile	N	-1.26919	99.8359
N-(5-chloro-2-methoxyphenyl)-2,4-dimethoxybenzamide	N	-1.26871	103.5585
5-phenyl-1-(2-thienyl)-2,4-pentadien-1-one	N	-1.26838	104.3058
4-[3-(2-thienyl)acryloyl]phenyl methanesulfonate	N	-1.26791	105.7281
2-oxo-2H-chromen-4-yl 3-phenylacrylate	N	-1.26728	95.1711
6-(phenylsulfanyl)-3-pyridinylamine	N	-1.26704	98.9003
2,5-diphenyl-4,6-pyrimidinediol	N	-1.26642	97.2686
2-[(4-bromophenyl)sulfanyl]-N-(4-chlorophenyl)acetamide	N	-1.2644	89.6471
3-propyl-7H-[1,2,4]triazolo[3,4-b][1,3,4]thiadiazine	N	-1.2644	95.0737
methyl 4-(phenylsulfanyl)-5-pyrimidinecarboxylate	N	-1.26434	118.3651
2-(2,5-dibutoxyphenyl)thiophene	N	-1.2639	87.9664
2-[(3-methylbenzoyl)amino]-N-[2-(4-morpholinyl)ethyl]benzamide	N	-1.26171	100.1661
4-hydroxy-3-(N-hydroxypropanimidoyl)-1-phenyl-5,6,7,8-tetrahydro-2(1H)-quinolinone	N	-1.26171	104.9764
[2-(1-azepanyl)-4-(4-methoxyphenyl)-1,3-thiazol-5-yl](phenyl)methanone	N	-1.26163	103.9444
(1,8,8-trimethyl-2,4-dioxo-3-azabicyclo[3.2.1]oct-3-yl)methyl 2-[1-(4-methoxyphenyl)ethylidene]hydrazinecarbodithioate	N	-1.26147	114.1921
1-(4-fluorophenyl)-3-(4-methoxyphenyl)-1,3-propanedione	N	-1.26141	93.2266
4-(cyclohexylamino)-2H-chromen-2-one	N	-1.26115	91.4981
6-(benzylamino)-1-methyl-2,4(1H,3H)-pyrimidinedione	N	-1.25925	90.3071
1,3-dicyclohexyl-6-(methylamino)-2,4(1H,3H)-pyrimidinedione	N	-1.25818	112.9434
2,2,2-trifluoro-N-(3-(2-oxo-2-phenylethyl)-1,3-thiazol-2(3H)-ylidene)acetamide	N	-1.25589	106.9036

1-(benzyloxy)-2(1H)-pyridinone	N	-1.25582	100.1631
N-(5-chloro-2-pyridinyl)-3-methylbenzamide	N	-1.25572	96.6433
S-(2-furylmethyl) 3-(4-ethoxyphenyl)-2-propenethioate	N	-1.25512	92.8669
S-ethyl 12-hydroxydodecanethioate	N	-1.25499	89.2182
6-amino-2-(benzylamino)-4(3H)-pyrimidinone	N	-1.25445	105.0912
4-chloro-2-phenyl-5-pyrimidinecarbonitrile	N	-1.2537	105.7929
1-(4-thiomorpholinylmethyl)-1,2-dihydro-3,6-pyridazinedione	N	-1.25368	108.1704
2-(2-cyclohexen-1-ylcarbonyl)-N-phenylhydrazinecarbothioamide	N	-1.25354	99.3654
2-({4-chloro-2-nitrophenyl}sulfanyl)cyclohexanone	N	-1.25273	86.705
N-ethyl-N-[2-(1H-indol-3-yl)ethyl]-N-methylamine	N	-1.24834	104.1816
4-(3-oxo-3-phenyl-1-propenyl)phenyl 3-phenylacrylate	N	-1.24833	101.4451
3-pyridinyl 4-chlorobenzenesulfonate	N	-1.24791	108.7822
2-[(4-chlorobenzyl)sulfanyl]-1-phenylethanone	N	-1.24729	89.0394
2-{4-[5-(butylsulfanyl)-2-thienyl]phenyl}-4,4-dimethyl-4,5-dihydro-1,3-oxazole	N	-1.24349	92.2307
2-(4-ethoxyphenyl)-4-oxo-4H-chromen-3-yl 2,4,6-trimethylbenzenesulfonate	N	-1.24315	95.4147
4-isopropoxy-6,8-dimethyl-7-methylene-7,8-dihydro-2-pteridinamine	N	-1.24189	89.5993
5-pentyl-2-[4-(4-pentylcyclohexyl)phenyl]-1,3-dioxane	N	-1.24153	89.8218
4-[(4-methoxybenzylidene)amino]-5-phenyl-4H-1,2,4-triazol-3-yl hydrosulfide	N	-1.24135	117.3019
2-(benzylamino)-N-(4-chlorophenyl)-2-thioxoacetamide	N	-1.23731	94.6394
5,6-dimethyl-2-phenyl-4-pyrimidinyl methyl ether	N	-1.237	101.2012
N-(4-fluorophenyl)-2-{[4-methyl-5-(trifluoromethyl)-4H-1,2,4-triazol-3-yl]sulfanyl}acetamide	N	-1.23669	94.0447
1,3-Dicarboethoxy-4-quinolizone	N	-1.23623	96.5093

1-(3-methylbenzoyl)piperidine	N	-1.23528	100.9013
N-(3,5-dimethylphenyl)-2-(dodecylsulfanyl)acetamide	N	-0.87262	86.1333
5-tridecyldihydro-2(3H)-furanone	N	-0.872059	111.9152
1-[4-(2-oxo-1(2H)-pyridinyl)butyl]-2(1H)-pyridinone	N	-0.870152	106.77
2-{[2-nitro-4-(trifluoromethyl)phenyl]sulfanyl}cyclohexanone	N	-0.86943	93.175
N-(3,5-dimethylphenyl)-2-(2-pyridinylsulfanyl)acetamide	N	-0.867471	104.272
N-[4-chloro-3-(trifluoromethyl)phenyl]pentanamide	N	-0.804454	100.5842
N-(4-methoxyphenyl)-2-{[5-(methylsulfanyl)-1,3,4-thiadiazol-2-yl]sulfanyl}acetamide	N	-0.80444	89.874
N-(1,3-benzodioxol-5-yl)-2-[(4-chlorophenyl)sulfanyl]acetamide	N	-0.803853	97.5036
4-phenyl-6-propoxy-2-pyrimidinylamine	N	-0.802879	136.894
N-(3,5-dimethylphenyl)-2-[(4,6-dimethyl-2-pyrimidinyl)sulfanyl]acetamide	N	-0.802391	132.043
5-hexyl-1,3,5-triazinane-2-thione	N	-0.802164	111.4603
N-[4-chloro-3-(trifluoromethyl)phenyl]-2-[(2-furylmethyl)amino]-2-thioxoacetamide	N	-0.802082	97.4488
2-{2-[4-(diethylamino)phenyl]vinyl}-1-methylpyridinium	N	-0.801638	85.5816
2-(1H-1,2,3-benzotriazol-1-yl)ethyl octyl sulfide	N	-0.800215	112.9332
2-(1,3-benzothiazol-2-ylsulfanyl)-N-(3-chlorophenyl)acetamide	N	-0.797797	112.607
4-(dodecyloxy)-1-naphthonitrile	N	-0.746725	109.9891
7-chloro-N-(2-{4-[2-(1-pyrrolidinyl)ethyl]-1-piperazinyl}ethyl)-4-quinolinamine	N	-0.746463	95.3273
2-[(4'-decyl[1,1'-biphenyl]-4-yl)oxy]propanamide	N	-0.746024	87.7184
N-(4,6-dimethyl-2-pyrimidinyl)[1,1'-biphenyl]-4-carboxamide	N	-0.745937	103.4845
N-(1,3-benzodioxol-5-yl)-2-[(4-methyl-4H-1,2,4-triazol-3-yl)sulfanyl]acetamide	N	-0.744815	107.2606
4-oxo-4H-pyrido[1,2-a]pyrimidin-2-yl benzoate	N	-0.742712	93.277

1-(3-chlorobenzyl)-4-(2-furoyl)piperazine	N	-0.74149 7	94.08 05
N-[2-chloro-5-(trifluoromethyl)phenyl]-2-[(4,6-dimethyl-2-p yrimidinyl)sulfanyl]acetamide	N	-0.74137 6	91.16 41
2-[(4,6-dimethyl-2-pyrimidinyl)sulfanyl]-N-(5-methyl-3-isox azolyl)acetamide	N	-0.74122	107.1 879
methyl 4-(3-phenyl[1,2,4]triazolo[3,4-b][1,3,4]thiadiazol-6-yl)phenyl ether	N	-0.73951 1	87.18 73
4-[3-(4-chlorophenyl)[1,2,4]triazolo[3,4-b][1,3,4]thiadiazol-6-yl]phenyl methyl ether	N	-0.73913 1	87.62 21
1-[4'-(octyloxy)[1,1'-biphenyl]-4-yl]-4-pentylcyclohexanol	N	-0.73826 5	95.08 51
5-amino-N-(1-ethyl-3-piperidinyl)-2-methoxybenzamide	N	-0.70925 1	93.78 76
2-(1-methyl-2-pyrrolidinylidene)-1-(2-thienyl)ethanone	N	-0.70778 9	90.92 6
4-methyl-3-(methylsulfanyl)-1,5-di(2-thienyl)-2-pentene-1,5-dione	N	-0.70758 8	123.0 39
1-(3-methoxyphenyl)-2-phenylethanol	N	-0.70720 2	103.7 542
4-(3,4,5-trimethoxybenzoyl)morpholine	N	-0.70666 5	134.0 27
(1,3-benzothiazol-2-ylsulfanyl)acetic acid	N	-0.70605 8	89.13 22
N-cyclopentyl-2-methylaniline	N	-0.70523 7	103.6 248
5-iodo-6-(octyloxy)-2-naphthoic acid	N	-0.70459 3	90.69 46
N~1~-{2-[(2-fluorophenyl)sulfanyl]benzyl}-N~1~,N~2~,N~2~-trimethyl-1,2-ethanediamine	N	-0.70427 1	101.5 513
N-(4-chlorophenyl)-2-{[5-(phenoxymethyl)-4-phenyl-4H-1,2,4-triazol-3-yl]sulfanyl}acetamide	N	-0.70181 7	113.3 134
3,5-dimethyl-1-phenylpiperidine	N	-0.69978	111.5 838
2-amino-N-(2-oxo-3-piperidinyl)benzamide	N	-0.69921 3	120.3 979
2-[(2-thiomorpholinylmethyl)sulfanyl]ethanamine	N	-0.69897	114.6 63
N-benzyl-N-{2-[(benzylamino)methyl]butyl}amine	N	-0.69878 6	96.80 74
N-(3-methylphenyl)benzenesulfonamide	N	-0.69849 2	119.4 04
(4-methoxy-2-pyridinyl)methyl	N	-0.69677	95.52

N-(3-fluorophenyl)imidothiocarbamate		2	57
N-[2-(dimethylamino)ethyl]-2-[(2-fluorophenyl)sulfanyl]-N-methylbenzamide	N	-0.64298 7	105.3 548
4-(benzyloxy)-2-methylquinazoline	N	-0.64241	98.63 05
3-(cyclohexylsulfanyl)-4-hydroxy-1-methyl-2(1H)-quinolino ne	N	-0.64221 8	119.6 649
N-(3,4-dichlorophenyl)-2-fluorobenzamide	N	-0.63962 4	109.7 099
3-(dibutylamino)propyl 4-amino-2,6-dimethylbenzoate	N	-0.63957 8	97.97 9
1-[2-(3-chlorodibenzo[b,e]thiepin-11(6H)-ylidene)ethyl]-4-m ethylpiperazine	N	-0.6138	106.1 977
N-(2,5-dimethoxyphenyl)-2-fluorobenzamide	N	-0.61379 7	88.35 08
4-tert-butyl-1-cyclohexene-1-carboxylic acid	N	-0.61215 7	89.52 84
2-[(3-bromo-5-chloro-2-hydroxybenzylidene)amino]-4,5,6,7-tetrahydro-1-benzothiophene-3-carbonitrile	N	-0.61200 4	103.1 945
N-[3-(2-chloro-6-fluoro-9H-thioxanthen-9-ylidene)propyl]-N,N-dimethylamine	N	-0.60171 9	115.4 829
N-{2-[2,5-dimethoxy-4-(pentylsulfanyl)phenyl]ethyl}-N,N-d imethylamine	N	-0.60157 9	115.3 472
N-cyclohexyl-N'-(3-ethoxyphenyl)urea	N	-0.60043 5	113.1 872
2-[6-(2-oxo-2-phenylethyl)-2-piperidinyl]-1-phenylethanone	N	-0.60029 2	98.95 34
5-(2-tert-butylanilino)-2,4-dimethyl-5-oxopentanoic acid	N	-0.57785 5	96.13 08
2-(4-morpholinylmethyl)-1H-benzimidazole	N	-0.57697 3	102.7 81
N-{4-[butyl(methyl)amino]phenyl}acetamide	N	-0.57633 6	105.2 055
5-methyl-2-phenyl-1,3-benzoxazole	N	-0.54003	102.3 472
N-(3-chlorophenyl)benzamide	N	-0.53939 9	101.5 784
1,5-di(2-furyl)-4-methyl-3-(methylsulfanyl)-2-pentene-1,5-di one	N	-0.53919 2	95.90 41
5-(dimethylamino)-1,2-diphenyl-3-pentanone	N	-0.53741	91.97 58
N-(2,4-dichlorophenyl)nicotinamide	N	-0.53737 9	87.00 37
N,N'-dibenzylurea	N	-0.53704	86.75

		2	77
N'-cyclohexylidene-2-(2,4-dichlorophenoxy)acetohydrazide	N	-0.53635<br>4	102.4<br>759
1-[6-methyl-1-(2-methylphenyl)-4-(methylsulfanyl)-2-thioxo-1,2-dihydro-3-pyridinyl]ethanone	N	-0.42056<br>9	107.3<br>434
2-[(4-chlorophenyl)sulfanyl]-N'-hydroxyethanimidamide	N	-0.41919<br>7	92.47<br>49
2-(1H-indol-3-yl)ethanol	N	-0.41914<br>3	114.1<br>14
1-methyl-2-[2-(phenylsulfanyl)phenyl]ethylamine	N	-0.41811<br>1	94.64<br>66
N-benzyl-N'-(4-methylphenyl)thiourea	N	-0.41507<br>2	101.9<br>867

*Data for the top 10 scoring compounds are highlighted in yellow .
